# Crystal Growth Engineering for Dendrite‐Free Zinc Metal Plating

**DOI:** 10.1002/adma.202510906

**Published:** 2025-09-30

**Authors:** Guifang Zeng, Sharona Horta, Qing Sun, Malik Dilshad Khan, Maria Ibáñez, Yuhang Han, Shang Wang, Longqiu Li, Lijie Ci, Yanhong Tian, Andreu Cabot

**Affiliations:** ^1^ Catalonia Institute for Energy Research (lREC) Sant Adrià de Besòs Barcelona 08930 Spain; ^2^ Zhengzhou Research Institute Harbin Institute of Technology Zhengzhou 450000 China; ^3^ State Key Laboratory of Precision Welding & Joining of Materials and Structures Harbin Institute of Technology Harbin 150001 China; ^4^ Institute of Science and Technology Austria (ISTA) Am Campus 1 Klosterneuburg 3400 Austria; ^5^ State Key Laboratory of Robotics and Systems Harbin Institute of Technology Harbin 150001 China; ^6^ ICREA Pg. Lluis Companys Barcelona 08010 Spain

**Keywords:** aqueous zinc‐ion battery, epitaxial growth, rare‐earth metal, screw dislocation growth, zinc anode

## Abstract

The practical implementation of aqueous zinc‐ion batteries (AZIBs) is limited by uncontrolled zinc (Zn) dendrite growth during anode plating, compromising both safety and cycle life. Typically, Zn plating proceeds via 2D growth along the six equivalent prismatic [$10\bar{1}0]$ directions of the hexagonal close‐packed (HCP) Zn lattice, forming hexagonal platelets that promote dendrite formation. Here, an effective electrolyte engineering strategy is presented using rare‐earth ions to regulate Zn plating. Combined multiscale experimental analyses and computational modeling reveal that these ions preferentially adsorb onto the prismatic {$10\bar{1}0$} facets, suppressing lateral epitaxial growth of the basal (0002) planes. This redirects Zn plating toward an apparent screw dislocation‐driven growth along the [0001] axis. The resulting growth pathway, together with randomly oriented Zn nucleation, yields dense, uniform, and dendrite‐free Zn layers with markedly improved cycling stability and high depth‐of‐discharge operation, thereby challenging the prevailing assumption that dendrite suppression requires (0002)‐oriented growth parallel to the substrate. This work provides new mechanistic insights into Zn plating dynamics and establishes a scalable strategy for stable, dendrite‐free Zn anodes in next‐generation AZIBs.

## Graphical abstract

This research employed the rare‐earth ion dysprosium (Dy) to modulate aqueous zinc (Zn) metal plating. Integrated multiscale experiments and computational modeling unveiled the preferential adsorption of Dy on specific crystal facets, which activated screw dislocation‐driven Zn growth. Consequently, dense, uniform, and dendrite‐free Zn plating is realized, delivering markedly enhanced cycling stability and enabling high depth‐of‐discharge during Zn plating/stripping.

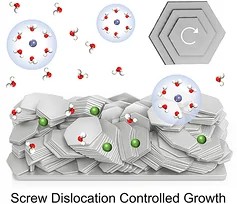

## 1 Introduction

In the global pursuit of energy security and environmental sustainability, the development of green, efficient, and durable energy storage technologies has become a critical priority.^[^ ^1^, ^2^, ^3^, ^4^ ^]^ Among emerging contenders, aqueous zinc‐ion batteries (AZIBs) have attracted significant attention for both front‐of‐the‐meter and behind‐the‐meter stationary energy storage due to their intrinsic safety,^[^ ^5^, ^6^, ^7^, ^8^, ^9^, ^10^, ^11^ ^]^ environmental benignity,^[^ ^12^, ^13^ ^]^ resource abundance,^[^ ^14^ ^]^ and low manufacturing cost.^[^ ^15^, ^16^ ^]^ However, the commercial viability of AZIBs is critically limited by the formation of zinc (Zn) dendrites during repeated cycling, which compromises anode stability, reduces cycle life, and poses serious safety risks.^[^ ^17^, ^18^, ^19^, ^20^, ^21^ ^]^

Electrolyte engineering has emerged as a powerful strategy for addressing dendrite formation by tuning the Zn plating/stripping behavior.^[^ ^22^, ^23^, ^24^ ^]^ In particular, the use of functional additives offers a simple and broadly compatible route to modulate interfacial processes.^[^ ^25^, ^26^, ^27^, ^28^ ^]^ Both organic and inorganic additives have been reported to suppress dendritic growth through various physicochemical mechanisms.^[^ ^29^, ^30^, ^31^, ^32^ ^]^ A frequently proposed explanation suggests that these additives facilitate preferential Zn plating in directions normal to [0001], leading to the formation of a basal (0002)‐oriented texture parallel to the substrate.^[^ ^33^, ^34^, ^35^ ^]^ However, this model assumes oriented Zn nucleation, which appears to contradict experimental observations.

Metal electroplating is governed by atomic‐scale surface processes such as adsorption, diffusion, and nucleation, all of which are influenced by crystallographic orientation and thermodynamic factors.^[^ ^36^ ^]^ Under ideal conditions, metal ions are reduced upon reaching the electrode surface, where they preferentially bind onto high‐energy sites such as step edges and lattice defects,^[^ ^37^ ^]^ promoting growth along specific crystallographic directions. In Zn, which crystallizes in a hexagonal close‐packed (HCP) structure, anisotropic surface energies play a central role in determining growth modes. In conventional aqueous electrolytes, the relatively high surface energy of the prismatic planes favours growth along the six equivalent [$10\bar{1}0]$ directions. This leads to the formation of hexagonal platelets with enlarged (0002) basal facets,^[^ ^38^ ^]^ As plating progresses, secondary nucleation at surface defects gives rise to additional Zn platelets with random orientations, ultimately resulting in vertically aligned dendritic structures that can penetrate the separator and cause short circuits.^[^ ^39^ ^]^

This thermodynamically driven 2D epitaxial growth of hexagonal platelets, combined with Zn's intrinsically low dislocation density, suppresses screw dislocation‐driven growth, a mechanism suggested to promote more uniform and compact Zn plating.^[^ ^39^ ^]^ Achieving a transition from epitaxial to dislocation‐mediated growth is therefore a critical step toward enabling long‐term cycling stability in Zn anodes. However, this transition remains underexplored within the framework of crystal growth theory in the context of AZIBs.

In this work, we introduce a simple yet effective strategy to modulate Zn plating by introducing rare‐earth metal ions into a conventional aqueous Zn electrolyte. Through integrated experimental analysis and first‐principles simulations, we demonstrate that these ions preferentially adsorb onto the six equivalent prismatic ($10\bar{1}0)$ facets of the HCP Zn lattice. This facet‐selective adsorption suppresses lateral growth of the (0002) basal planes, thereby inhibiting 2D epitaxial expansion and promoting screw dislocation‐driven plating along the [0001] direction. This shifted growth pathway, together with our evidence of randomly oriented Zn nucleation, facilitates the formation of dense, uniform, and dendrite‐free Zn layers exhibiting outstanding cycling reversibility and structural stability. Altogether, this work establishes a new electrolyte design paradigm that deepens the mechanistic understanding of facet‐directed metal plating and provides a scalable approach for the development of stable, high‐performance AZIBs.

## 2 Results and Discussion

A 2 m ZnSO_4_ aqueous solution, widely adopted as a standard electrolyte in AZIBs, was used as the baseline electrolyte in this study and is referred to as ZS. To investigate the impact of rare‐earth ion modification, dysprosium chloride (DyCl_3_) was employed as an electrolyte additive. Dy^3+^‐modified electrolytes were prepared by dissolving 0.03, 0.05, 0.08, and 0.1 m DyCl_3_ into the ZS solution (Figure S1, Supporting Information), being denoted as DZS‐0.03, DZS‐0.05, DZS‐0.08, and DZS‐0.1. The DZS‐0.05 presents the best ionic conductivity (Figure S2, Supporting Information), which is expected to alleviate concentration polarization at the Zn anode interface.^[^ ^40^ ^]^ To evaluate the Zn plating/stripping performance in different electrolytes, symmetric Zn||Zn cells were assembled. As shown in Figure S3 (Supporting Information), the Zn||Zn cell with DyCl_3_ additives exhibits high reversibility and a stable capacity of 1 mAh g^−1^ at a current density of 2 mA cm^−2^. In contrast, the cell using ZS electrolyte sustains cycling for merely 47 h. Increasing the DyCl_3_ concentration to 0.05 m yields the longest cycle life of 450 h with a low voltage hysteresis. However, further increasing the concentration beyond 0.05 m somehow raises the voltage hysteresis. Based on these results, the electrolyte containing 0.05 m DyCl_3_ was selected as the optimal concentration for subsequent experiments in this study.

**Figure 1:**
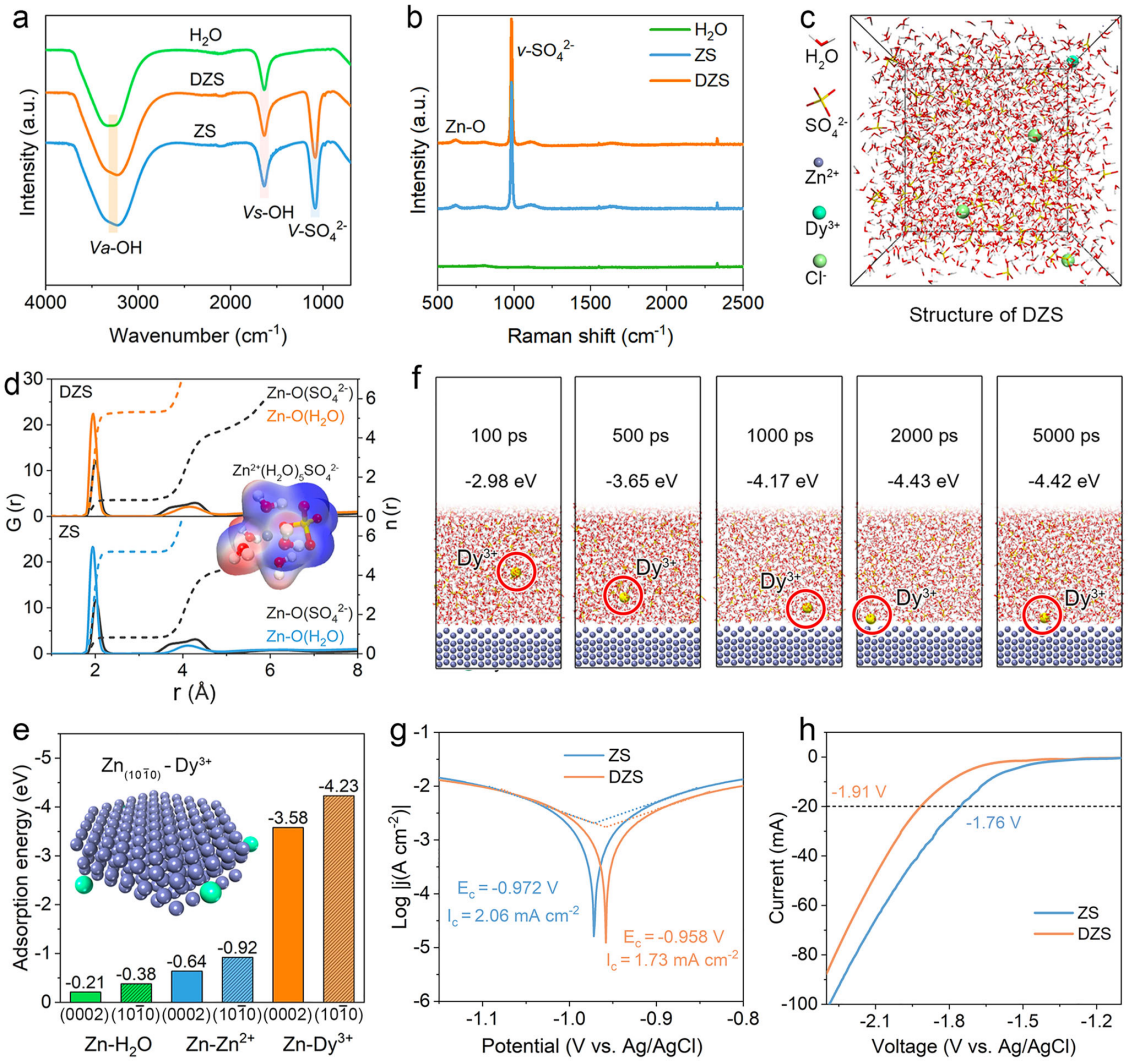
a) FTIR and b) Raman spectra of ZS and DZS electrolytes. c) Atomistic model of the DZS electrolyte used in MD simulations. d) Radial distribution function (G, solid line) and coordination number profile (n, dashed line) for Zn^2+^ solvation in the DZS electrolyte. e) Adsorption energies of Zn‐H_2_O, Zn‐Zn^2+^, and Zn‐Dy^3+^ complexes. f) Snapshot of the MD simulation illustrating Dy^3+^ interactions at the DZS electrolyte/Zn anode interface. g) Tafel plots. h) LSV curves.

The introduction of electrolyte additives often alters the internal solvation structure, potentially leading to a cascade of complex effects on electrochemical behavior. To assess the impact of the added 0.05 m Dy^3+^ on the Zn^2+^ solvation environment, Fourier transform infrared (FTIR) and Raman spectroscopies combined with molecular dynamics (MD) simulations were employed. As shown in **Figures** **1**a and S4 (Supporting Information), the FTIR spectra revealed negligible differences in the vibrational modes associated with SO_4_^2−^ and OH^−^ species between the ZS and DZS electrolytes, indicating that the primary solvation shell of Zn^2+^ remains largely unaffected upon Dy^3+^ addition within the bulk electrolyte. Raman spectroscopy further corroborated this observation, with the characteristic peaks attributed to Zn‐O and SO_4_^2−^ interactions displaying no appreciable shifts following Dy^3+^ incorporation (Figure 1b).

Additional molecular‐level insights were obtained from MD simulations (Figures 1c; S5, Supporting Information). Figure 1d presents the correlations between the radial distribution function G(r), the coordination number n(r), and the corresponding interatomic distance r across different electrolyte models. Zn^2+^ maintains coordination with ≈6 water molecules, preserving the canonical [Zn(H_2_O)_6_]^2+^ solvation structure in both ZS and DZS systems. Collectively, these results confirm that the introduction of Dy^3+^ at low concentration does not significantly perturb the Zn^2+^ solvation environment in the bulk electrolyte.

Owing to its high charge density, Dy^3+^ introduced into the ZnSO_4_ electrolyte exhibits a strong propensity to migrate toward the Zn metal anode and stably adsorb onto its surface. Density functional theory (DFT) calculations show the adsorption energy of Dy^3+^ on Zn is significantly more negative than that of either Zn–H_2_O or Zn–Zn^2+^ interactions (Figure 1e), indicating a clear thermodynamic preference for Dy^3+^ adsorption. This preferential binding effectively displaces water molecules and Zn^2+^ from direct contact with the Zn surface. MD simulations further confirmed the strong interfacial affinity of Dy^3+^. Figure 1f shows that Dy^3+^ in the ZS electrolyte reached the Zn anode at ≈2000 ps and then migrated along its surface over the following 3000 ps, lowering the system energy and stabilizing the configuration. This suggests that Dy^3+^ tends to accumulate at the Zn/electrolyte interface, forming a Dy^3+^‐enriched electric double layer. This interfacial structure is expected to suppress parasitic reactions such as hydrogen evolution (HER) and the formation of insoluble byproducts like Zn_4_SO_4_(OH)_6_·xH_2_O, both of which, alongside dendrite formation, compromise the cycling stability of Zn anodes.

The effect of Dy^3+^ accumulation at the Zn surface on interfacial ion transport and desolvation behavior was systematically investigated. Due to its high charge density and strong electrostatic field, a high local concentration of Dy^3+^ at the Zn surface can disrupt the primary solvation shell of Zn^2+^, thereby facilitating the removal of coordinated solvent molecules and accelerating the desolvation process. Arrhenius analysis of the interfacial impedance, measured by electrochemical impedance spectroscopy (EIS) at different temperatures (Figure S6, Supporting Information), and the activation energy associated with desolvation was determined from the Arrhenius equation (Figure S7, Supporting Information):^[^ ^41^ ^]^

(1) $$\frac{1}{R_{\mathrm{ct}}}=\mathrm{Aexp}\left(-\frac{E_{a}}{\mathrm{RT}}\right)$$

where R_ct_ denotes the charge transfer resistance, A the frequency factor, E_a_ the activation energy, R stands for the gas constant, and T the temperature. This result shows that the DZS electrolyte exhibits only a marginal reduction in the activation energy for Zn^2+^ desolvation (35.2 Kj mol^−1^) compared to the Dy^3+^‐free ZS system (38.4 kJ mol^−1^). Although such small variations have been used as qualitative probes in prior studies, the difference here is considered to fall within experimental uncertainty and is thus not considered significant.

The impact of Dy^3+^ on Zn anode stability was further assessed via Tafel polarization analysis (schematic diagram of extrapolation method in Figure S8, Supporting Information). The addition of Dy^3+^ induces a positive shift in the corrosion potential (Figure 1g), from −0.972 V (ZS) to −0.958 V (DZS), corresponding to ≈14 mV. Meanwhile, the corrosion current density is markedly reduced from 2.06 to 1.73 mA cm^−2^, i.e., 16%, indicating improved electrochemical stability and corrosion resistance. To further validate these results and exclude potential experimental errors, we analyzed three additional sets of tests (Figure S9a–c and Table S1, Supporting Information). Systematic differences were observed when comparing DZS and ZS electrolytes: i) the corrosion potential shifted to more positive values for the DZS electrolyte, and ii) the absolute current within this window decreased in magnitude for DZS. Together, these effects consistently yielded clearly and significantly lower corrosion current density for DZS electrolytes compared with ZS, confirming that the addition of Dy^3+^ has a genuine effect. To further cross‐check the corrosion current, we independently confirmed its reliability using the Stern–Geary method (Table S2, see detailed Experimental Section in Supporting Information).^[^ ^42^ ^]^ Moreover, increasing the additive concentration to 0.1 m (DZS‐0.1) further decreases the corrosion current density to 0.42 mA cm^−2^ and shifts the corrosion potential to −0.936 V (Figure S9d and Table S1, Supporting Information), thereby corroborating the enhanced corrosion resistance imparted by Dy^3+^.

These changes are meaningful in the context of electrochemical corrosion studies: i) a positive shift in corrosion potential reflects a more thermodynamically stable electrode/electrolyte interface, and ii) a decrease in corrosion current density indicates suppressed corrosion kinetics. Taken together, these changes suggest that the DZS electrolyte confers enhanced electrochemical stability and improved resistance against Zn anode corrosion.

Furthermore, linear sweep voltammetry (LSV) was employed to evaluate the HER activity. The DZS electrolyte displays a more negative HER onset potential (Figure 1h), suggesting effective suppression of water reduction. This inhibition is attributed to Dy^3+^ modifying the interfacial solvation environment by promoting Zn^2+^ desolvation at the Zn‐electrolyte interface, which in turn decreases the local concentration of free protons and raises the energy barrier for proton reduction, thereby retarding HER kinetics.

While Dy^3+^ adsorption on the Zn surface might help suppress parasitic reactions, its presence at the electrode–electrolyte interface raises concerns regarding potential electrochemical reduction. However, LSV reveals that Dy^3+^ exhibits a plating potential ≈0.46 V more positive than that of Zn^2+^, indicating that Dy^3+^ is thermodynamically unlikely to undergo electrochemical reduction under the applied operating conditions (Figure S10, Supporting Information). Furthermore, X‐ray Photoelectron Spectroscopy and Energy Dispersive Spectrometer analyses were performed on the cycled Zn foil to detect the presence of Dy. As shown in Figures S11–S13 (Supporting Information), no Dy signal is observed, indicating that Dy^3+^ is neither reduced nor involved in any side reactions. The electrochemical inertness of Dy^3+^ ensures the long‐term stability and durability of the DZS electrolyte during extended cycling.

**Figure 2:**
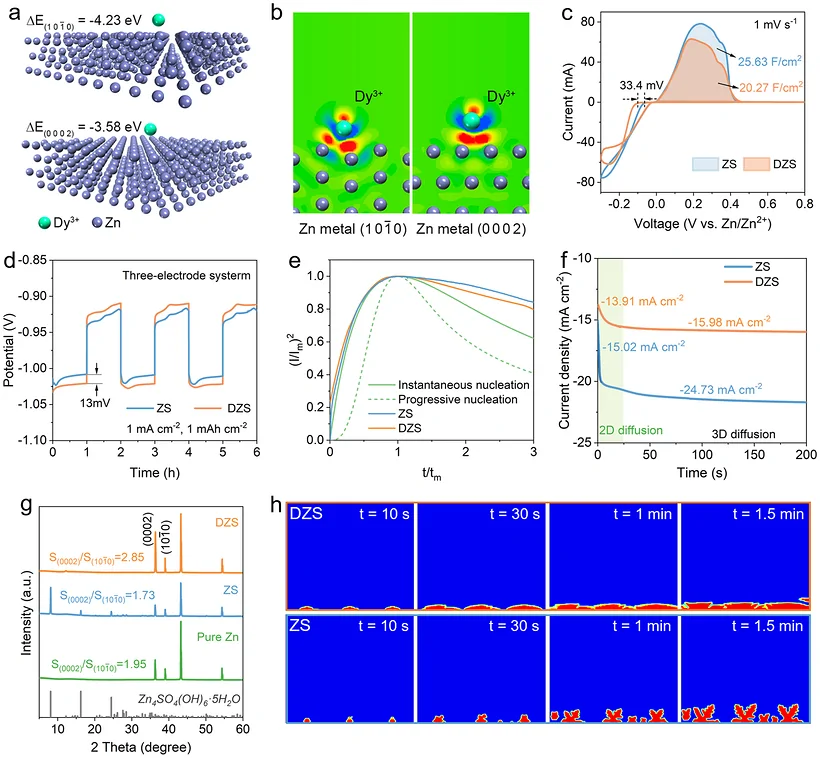
a) Atomic models of the Zn ($10\bar{1}0$) and (0002) facets with the corresponding Dy^3+^ adsorption energies. b) DFT‐calculated electronic charge density distribution upon Dy^3+^ adsorption on Zn surfaces. c) CV curves in ZS and DZS electrolytes, highlighting the differential overpotential and the stripping charge. d) GCD profiles in a 3‐electrode system. e) Comparison of experimental dimensionless transients with theoretical 3D nucleation models. f) CA test of Zn||Zn symmetric cells at −200 mV overpotential. g) XRD patterns and the corresponding values of S_(0002)_/$S_{\left(10\bar{1}0\right)}$. h) Phase‐field simulation results of the Zn plating in ZS and DZS electrolytes.

Given the intrinsically anisotropic nature of Zn's HCP crystal structure, the adsorption behavior of Dy^3+^ is expected to differ across various crystallographic facets. During Zn electroplating, the low energy basal (0002) and six crystallographically equivalent prismatic ($10\bar{1}0$) facets are predominantly exposed (Figure S14a,b, Supporting Information). To evaluate facet‐dependent interactions, DFT calculations were performed to determine the adsorption energies of Dy^3+^, Zn^2+^, and H_2_O on both the ($10\bar{1}0$) and (0002) surfaces (Figure S15, Supporting Information). As shown in **Figure** **2**a,b, Dy^3+^ exhibits significantly stronger adsorption on the prismatic ($10\bar{1}0$) surface (−4.23 eV) compared to the basal (0002) plane (−3.58 eV). This facet‐specific Dy^3+^ adsorption may preferentially suppress Zn growth along prismatic facets relative to the basal plane, as depicted schematically in Figure S16 (Supporting Information).

The partial Dy^3+^ surface coverage might modulate nucleation and crystal growth kinetics, ultimately impacting electrochemical performance. Cyclic voltammetry (CV) measurements show the DZS system displays a significantly higher Zn nucleation overpotential, with an increase of 33.4 mV relative to ZS (Figure 2c), confirming competitive interfacial adsorption between Zn^2+^ and Dy^3+^ at the Zn electrode surface. This competition was further quantified by analyzing the anodic stripping charge, which revealed a marked reduction in stripping coulombs in the presence of Dy^3+^, corroborating that Dy^3+^ perturbs Zn nucleation by occupying active sites on the electrode surface. Galvanostatic charge/discharge (GCD) profiles further support this conclusion. The DZS electrolyte exhibits a higher overpotential than the ZS counterpart (Figure 2d), reflecting retarded Zn plating kinetics under constant current conditions.^[^ ^43^ ^]^ Given the previously discussed computational findings, which show preferential Dy^3+^ adsorption on the ($10\bar{1}0)$ prismatic facets, it is reasonable to assume that this kinetic hindrance primarily affects 2D epitaxial growth of the (0002) basal planes.

Chronoamperometric (CA) measurements at −1.25 V were carried out to elucidate the nucleation dynamics of Zn metal. According to the Scharifker–Hills model, Zn nucleation can proceed via two primary pathways: i) 3D instantaneous nucleation (3D‐IN), where all nuclei form simultaneously at the onset of plating, and ii) 3D progressive nucleation (3D‐PN), characterized by the continuous emergence of new nuclei over time.^[^ ^44^, ^45^ ^]^ These mechanisms are described by the following theoretical expressions:

(2) $$3D-\mathrm{IN}:{\left(I/I_{m}\right)}^{2}=1.9542{\left(t/t_{m}\right)}^{-1}{\left\{1-\exp\left[-1.2564\left(t/t_{m}\right)\right]\right\}}^{2}$$

(3) $$3D-\mathrm{PN}:{\left(I/I_{m}\right)}^{2}=1.2254{\left(t/t_{m}\right)}^{-1}{\left\{1-\exp\left[-2.3367{\left(t/t_{m}\right)}^{2}\right]\right\}}^{2}$$

where *I* is the current at time *t*, *I_m_* is the peak current, and *t_m_* is the time at which this peak occurs. As illustrated in Figures 2e and S17 (Supporting Information), the experimental data from both ZS and DZS electrolytes closely match the 3D‐IN profile, indicating that Zn nucleation proceeds via an instantaneous mode in both cases. This suggests that the introduction of Dy^3+^ does not alter the fundamental electrocrystallization mechanism of Zn, but rather modulates subsequent growth dynamics.

Additional CA measurements at an overpotential (−200 mV) were conducted to investigate how Dy^3+^ incorporation influences Zn growth. As shown in Figure 2f, in the ZS electrolyte, a prolonged 2D diffusion regime is observed, with current density steadily increasing beyond 200 s, indicative of persistent epitaxial growth and surface diffusion during the early stages of nucleation. In contrast, the DZS electrolyte exhibits an attenuated current with a rapid transition from 2D to stable 3D diffusion within just 24 s, evidenced by a current plateau ≈15.98 mA cm^−2^. This behavior suggests uniform Zn plating governed by volumetric rather than surface‐limited growth dynamics in DZS,^[^ ^38^ ^]^ highlighting the critical role of Dy^3+^ in modulating Zn growth kinetics.

The crystalline structure of plated Zn was further investigated to uncover the structural modifications induced by Dy^3+^. X‐ray diffraction (XRD) analysis of cycled Zn anodes (Figure 2g) reveals that samples cycled in the ZS electrolyte display prominent diffraction peaks corresponding to the Zn_4_SO_4_(OH)_6_·5H_2_O byproduct, indicating extensive parasitic side reactions. In contrast, Zn anodes cycled in the DZS electrolyte exhibit minimal byproduct formation, underscoring the superior corrosion‐inhibiting effect imparted by Dy^3+^.

Additionally, a comparative analysis of the (0002) to ($10\bar{1}0$) and (0002) to ($10\bar{1}1)$ The diffraction peak integrated intensity (peak area) ratio was performed. S_(0002)_/$S_{\left(10\bar{1}0\right)}$ and S_(0002)_/$S_{\left(10\bar{1}1\right)}$ reveal notable electrolyte‐dependent growth behavior (Figures 2g; S18, Supporting Information). In pristine Zn, the S_(0002)_/$S_{\left(10\bar{1}0\right)}$ and S_(0002)_/$S_{\left(10\bar{1}1\right)}$ ratios are 1.95 and 0.4, respectively. After cycling in the ZS electrolyte, they decrease to 1.73 and 0.38, respectively, suggesting a preferential 2D growth along the [$10\bar{1}0$] directions, which is consistent with the lateral expansion of the (0002) basal plane typical of dendritic morphology. Conversely, cycling in the DZS electrolyte results in a substantial increase in this ratio to 2.85 and 0.85, indicating a pronounced preferential growth along the [0001] direction. This shift in crystallographic texture suggests that Dy^3+^ promotes dendrite‐free Zn plating, which is tentatively attributed to a growth mechanism dominated by screw dislocations, favoring compact, uniform plating over dendritic propagation.

Notably, while the ($10\bar{1}1$) plane exhibits a relatively strong Bragg signal, it is not further considered here because, under conventional electroplating conditions, Zn tends to form a HCP crystal structure. A key feature of this structure is the orientation relationship between the basal (0002) plane and the six equivalent prismatic ($10\bar{1}0$) planes, which are perpendicular to each other and together define the characteristic hexagonal platelet morphology. From a thermodynamic perspective, the (0002) plane possesses the lowest surface energy and free energy, and thus preferentially nucleates and grows during plating, becoming the dominant surface for epitaxial growth. Correspondingly, the six equivalent ($10\bar{1}0$) planes are typically exposed as the sidewalls of the crystal.

**Figure 3:**
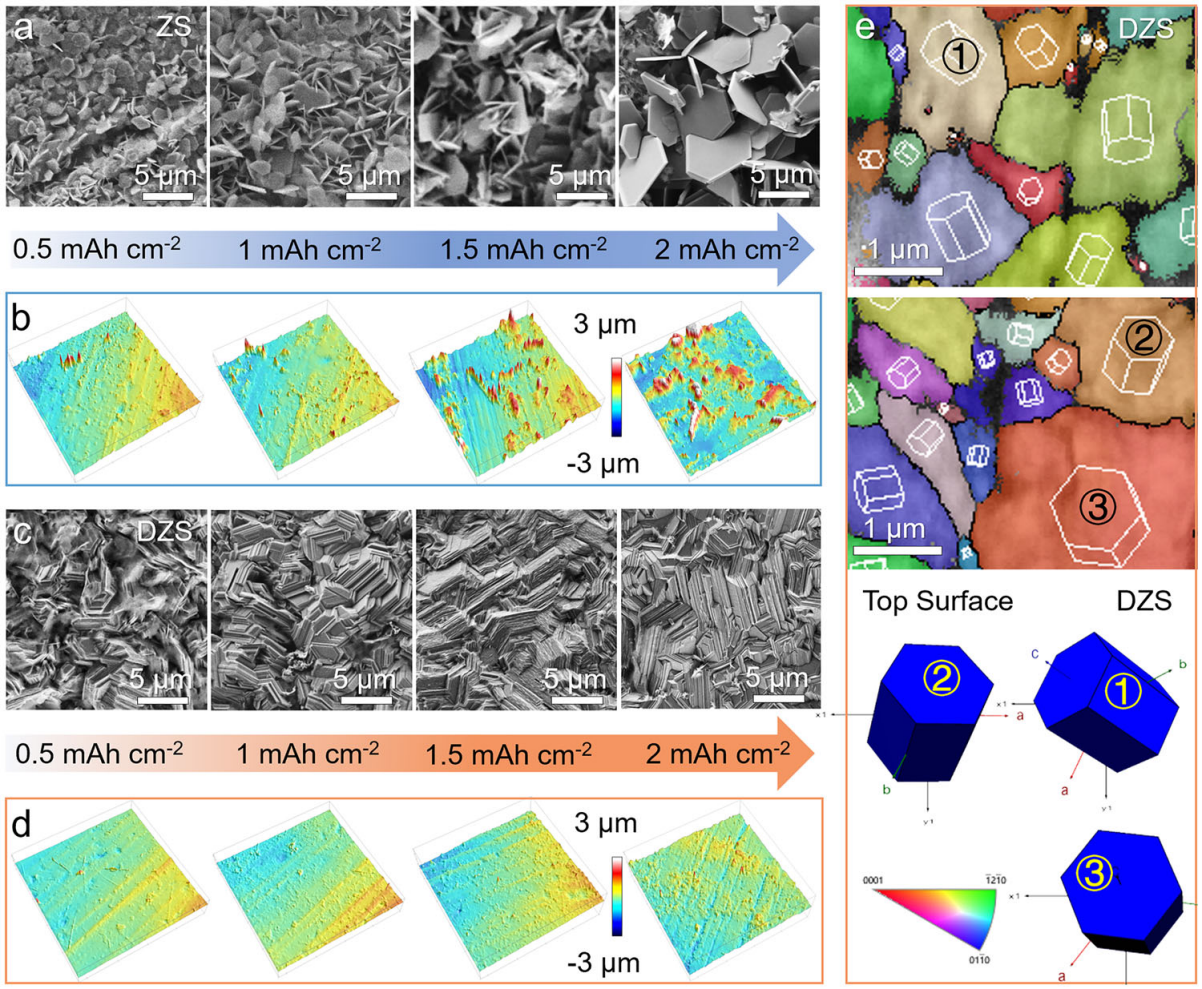
Analysis of the morphological evolution of plated Zn. a–d) SEM images a,c) and 3D surface profiles b,d) of Zn plated with a fixed capacity from 0.5 to 2 mAh cm^−2^ in ZS a,b) and DZS c,d) electrolytes. e) EBSD IPFs of the Zn anode plated in DZS at 2 mAh cm^−2^ and examples of the corresponding crystal orientations.

In this context, although the ($10\bar{1}1$) plane is crystallographically possible within the hexagonal system, it is not significantly exposed in the platelet morphology formed during electroplating (as shown in **Figure** **3**a). This is because the formation energy of the ($10\bar{1}1$) plane is relatively high, making it unfavorable to appear as a major external surface under standard conditions. Therefore, in experimentally observed morphologies, only the (0002) and the six equivalent ($10\bar{1}0$) planes are typically visible, while the ($10\bar{1}1$) plane is absent. In other words, the ($10\bar{1}1$) plane does not play a decisive role in the overall plating dynamics and morphological evolution.

Building on the preceding electrochemical analyses, we conducted phase‐field simulations to investigate the morphological evolution of Zn plating in the two electrolytes: ZS and DZS (Figure 2h). The results reveal that in the ZS electrolyte, Zn initially nucleates nearly simultaneously across the surface and gradually develops into hexagonal plates. As these plates expand to a certain size, their edges act as favorable sites for secondary nucleation, giving rise to additional hexagonal domains with varied orientations. This leads to the formation of interlaced, dendritic morphologies. In contrast, Zn plating in the DZS electrolyte proceeds via a stepwise, layer‐by‐layer growth behavior. Over time, adjacent grains merge, resulting in a more compact and uniform Zn‐plated layer.

To gain deeper insights into the Zn growth behavior during electroplating, the surface morphology of Zn plating formed in ZS and DZS electrolytes was examined using SEM and confocal microscopy. The pristine, polished Zn substrate displays a relatively smooth surface, as shown in Figures S19 and S20 (Supporting Information). In contrast, Zn plated from the ZS electrolyte develops the typical complex, interwoven morphology composed of hexagonal platelet‐like dendrites (Figure 3a,b), indicative of 2D epitaxial growth along the relatively higher energy [$10\bar{1}0$] crystallographic directions. Increasing the plating capacity to 2 mAh cm^−2^ leads to a more pronounced network of loosely packed, porous hexagonal structures. Notably, a similar dendritic morphology is observed when Zn is plated onto Cu foil (Figure S21a, Supporting Information).

In sharp contrast, Zn plated from the DZS electrolyte exhibits a compact and densely packed surface, free of dendritic features (Figure 3c,d). This dendrite‐free morphology is also reproduced when Zn is electroplated onto Cu foil (Figure S21b, Supporting Information), confirming the generality of the Dy^3+^‐induced suppression effect. The resulting surface displays a stepped topography with discernible hexagonal facets. While such features are often simplistically ascribed to a (0002)‐oriented growth with the basal planes aligned parallel to the substrate,^[^ ^46^, ^47^ ^]^ this assumption implies a uniform crystal orientation that would result in a tightly packed, honeycomb‐like hexagonal array. However, our observations display sporadic hexagonal facets oriented in multiple directions, a commonly reported yet frequently overlooked phenomenon in structural interpretations.

To probe the underlying crystallographic orientations, electron backscatter diffraction (EBSD) was performed on the morphology of the Zn anode plated at 2 mAh cm^−2^ (Figure 3e). The EBSD inverse pole figure (IPF) reveals a polycrystalline architecture composed of multiple grains with considerable orientation variance. This finding contradicts the hypothesis of uniform (0002)‐oriented growth, which would manifest in the EBSD IPF as consistent orientation across all grains.

**Figure 4:**
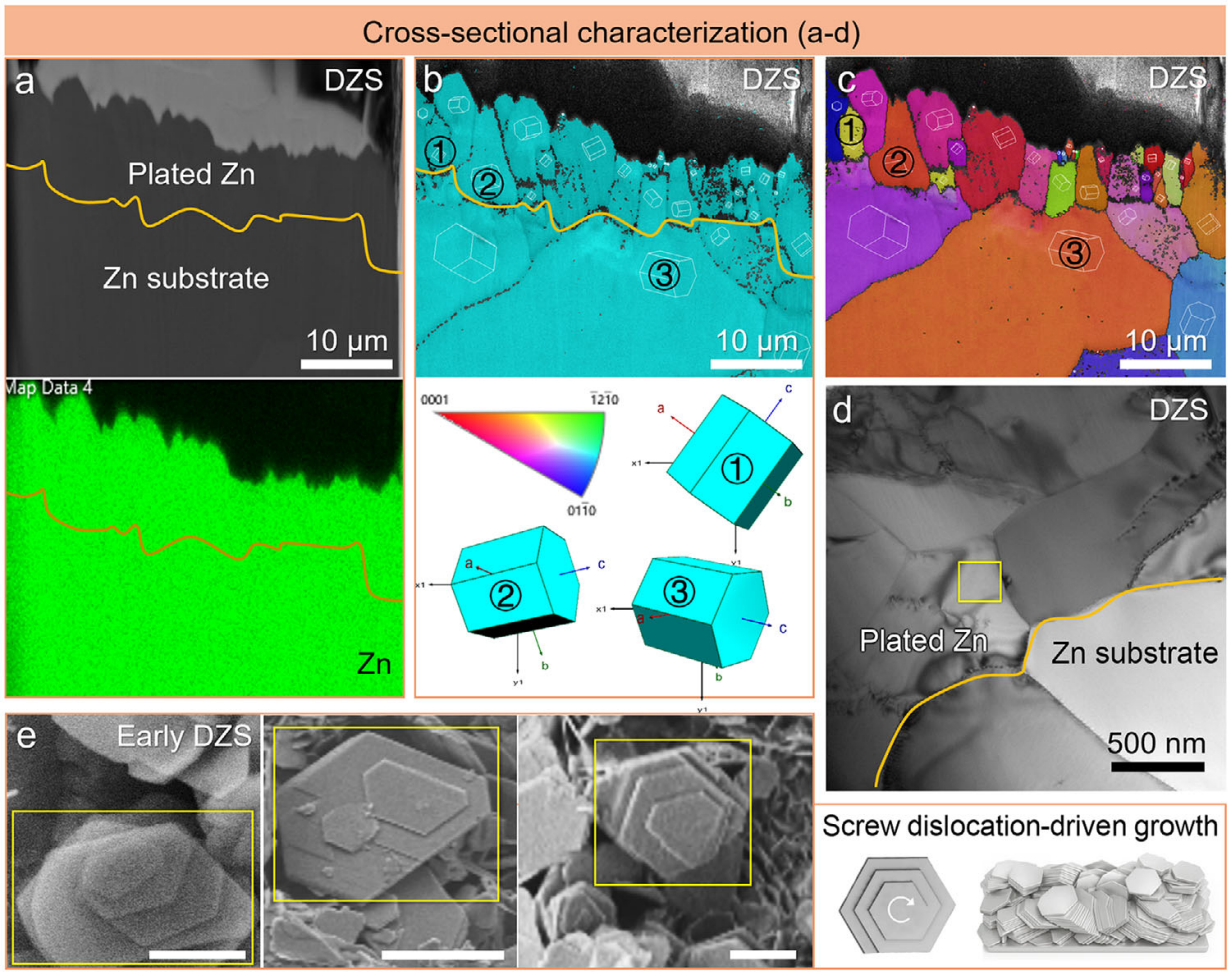
a) Cross‐section SEM image and corresponding Zn elemental mapping, b) crystallographic orientation distribution, c) EBSD IPF, and d) TEM image of plated layer in DZS electrolyte. e) SEM image showing srew dislocation‐driven growth in anodes obtained at 1 mA cm^−2^ with a capacity of ≈0.1 mAh cm^−2^ and the corresponding schematic illustration of this mechanism (scale bars: 5 µm).

To further elucidate the microstructural characteristics of Zn plated in the DSZ case, a sample cross‐section was prepared using a focused ion beam (FIB, Figure S22, Supporting Information). The cross‐sectional SEM image and corresponding Zn elemental mapping reveal that both the plated Zn layer and the underlying substrate exhibit a dense and pore‐free morphology (**Figure** **4**a). Crystallographic orientation analysis (Figures 4b,c; S23, Supporting Information) reveals that the Zn substrate displays a polycrystalline structure with relatively large grain sizes, while the electroplated Zn layer also exhibits a polycrystalline architecture but with significantly finer grains.

Transmission electron microscopy (TEM) and high‐resolution TEM (HRTEM) analyses further corroborate the polycrystalline morphology of the plated layer (Figures 4d; S24, Supporting Information) and confirm that each apparent grain retains its single‐crystalline internal structure. In sharp contrast to the ZS group (Figure S25, Supporting Information), the FIB‐prepared sample shows the polycrystalline substrate surface covered with flake‐like Zn plates, which partially crack during sample preparation due to their thinness. These results indicate that the step‐like surface morphology observed in Figure 3c originates from the compact assembly of multiple Zn grains with distinct orientations. Each parallel terrace segment corresponds to a single crystal grain with a random orientation, contradicting the widely accepted interpretation in prior reported literature that attributes such morphology to the (0002) planes growing all parallel to the substrate.^[^ ^33^, ^34^, ^35^ ^]^

Additionally, in the early stages of Zn plating in the DZS electrolyte, flake‐like structures are observed (Figures 4e; S26a, Supporting Information), exhibiting characteristic features of screw dislocation‐driven growth (Figure S26a–c, Supporting Information). Besides, when the concentration of DyCl_3_ in the electrolyte is drastically increased tenfold to 0.5 m, Zn electroplating clearly follows the [0001] crystallographic orientation,^[^ ^48^ ^]^ resulting in the growth of hexagonal nanowires (Figure S27a–c, Supporting Information).

Building on the preceding results, we conclude that in the Dy^3+^‐modified electrolyte, Zn nucleates on the substrate with random crystallographic orientations. The preferential adsorption of Dy^3+^ onto the prismatic ($10\bar{1}0$) facets, which constitute the lateral edges of the (0002) terraces, restricts 2D epitaxial growth, thereby shifting the growth regime toward step‐propagation mechanisms, potentially including a screw‐dislocation–mediated mode. As this growth proceeds, each Zn grain develops a stepped morphology and gradually merges with neighboring grains, yielding a densely packed, pore‐free polycrystalline Zn layer. Notably, this growth mechanism is not exclusive to Dy^3+^ regulation but may also be relevant to Zn morphologies influenced by other heteroionic electrolyte additives.

**Figure 5:**
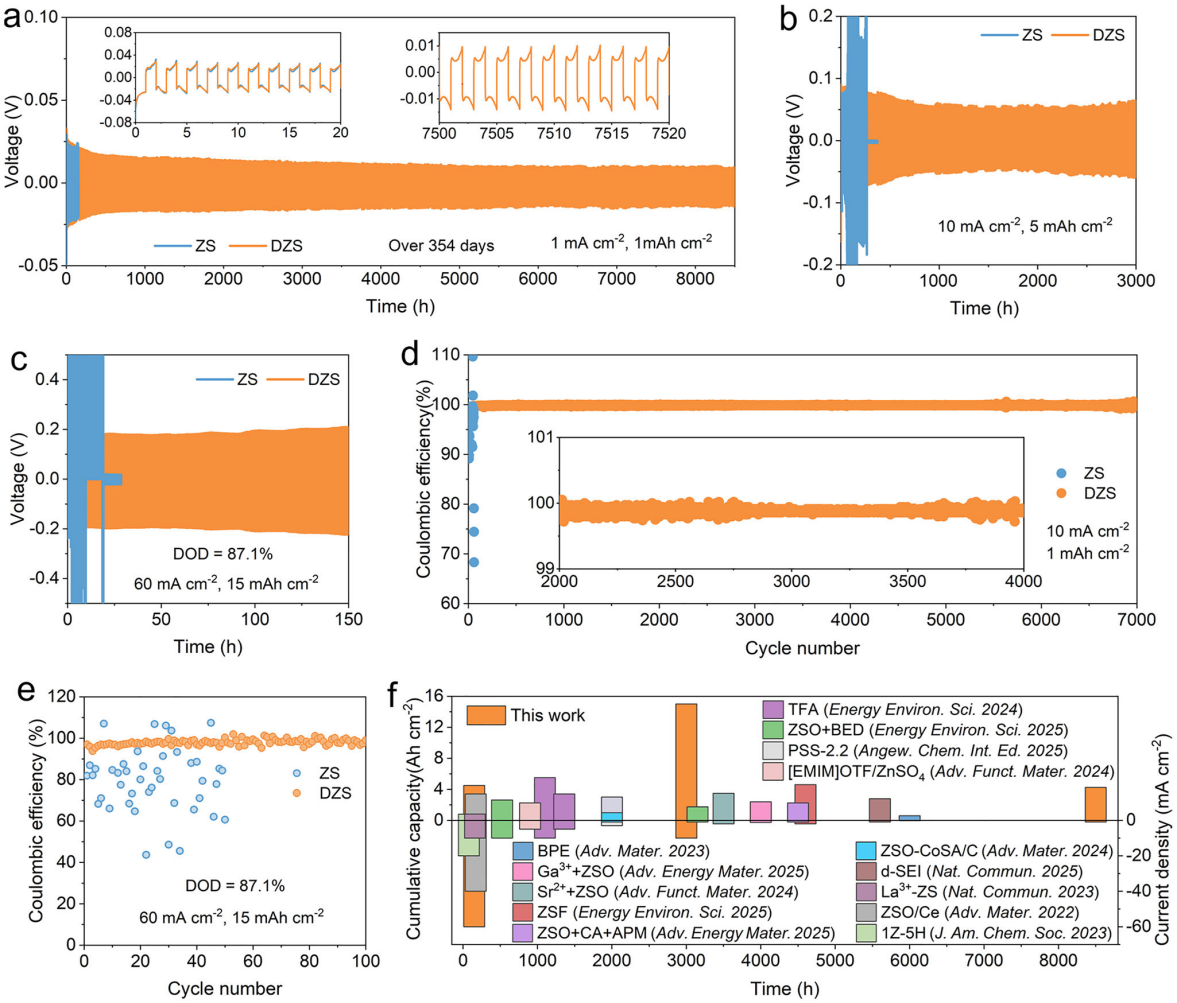
Electrochemical performance of Zn anodes in ZS and DZS electrolytes. a–c) Galvanostatic Zn plating/stripping profiles in Zn||Zn cells at 1 mA cm^−2^ with a capacity of 1 mAh cm^−2^ a), 10 mA cm^−2^ with a capacity of 1 mAh cm^−2^ b), and 60 mA cm^−2^ with a capacity of 15 mAh cm^−2^ c). d,e) CE of Zn||Cu cells at 10 mA cm^−2^ with a capacity of 1 mAh cm^−2^ d) and 60 mA cm^−2^ with a capacity of 15 mAh cm^−2^ e). f) Benchmarking of cyclic reversibility performance against recently reported Zn‐based systems.^[^ ^8^, ^17^, ^49^, ^50^, ^51^, ^52^, ^53^, ^54^, ^55^, ^56^, ^57^, ^58^, ^59^, ^60^ ^]^

To assess the influence of Dy^3+^ on electrochemical performance, specifically the stability of Zn plating and stripping, Zn||Zn symmetric cells were subjected to GCD cycling in both ZS and DZS electrolytes. As shown in **Figure** **5**a, the symmetric cell employing the DZS electrolyte exhibits outstanding long‐term stability, operating continuously for over 8500 h (≈354 days) at a current density of 1 mA cm^−2^ with a cutoff capacity of 1 mAh cm^−2^. This corresponds to a cumulative plated capacity (CPC) of 4.25 mAh cm^−2^, outperforming most state‐of‐the‐art Zn‐based systems reported to date (Table S3, Supporting Information). In sharp contrast, the cell using the ZS electrolyte experiences a sudden voltage drop and fails after only 234 h due to dendrite‐induced short‐circuiting. Even under more demanding conditions, 5 mAh cm^−2^ at 10 mA cm^−2^, the DZS‐based symmetric cell maintains stable operation for 3000 h (≈125 days), whereas the ZS counterpart fails within just 133 h (Figure 5b). These results demonstrate the critical role of Dy^3+^ in stabilizing Zn anodes by effectively suppressing dendrite formation and significantly extending the cycle life of AZIBs.

In laboratory‐scale studies, Zn anodes are often employed in large excess, resulting in electrochemical performance metrics that may not accurately reflect realistic device‐level conditions. To address this limitation, the depth‐of‐discharge (DOD) is a critical parameter for assessing the practical utilization efficiency of Zn anodes. In this work, we utilized a symmetric cell configuration incorporating an ultrathin Zn anode (30 µm) and operated at a high DOD of 87.1% to rigorously evaluate electrochemical stability. Under these stringent conditions, the symmetric cell with the DZS electrolyte demonstrated robust cycling stability for 150 h at an ultrahigh current density of 60 mA cm^−2^ (Figure 5c). In contrast, the cell with the ZS electrolyte exhibited premature failure, accompanied by a pronounced increase in overpotential.

Coulombic efficiency (CE) is another critical metric for evaluating the reversibility of Zn plating/stripping, as it quantifies the ratio of stripped Zn to the amount previously plated in each cycle. The CE performance of Zn||Cu cells with a 1‐h plating duration at 2 mA cm^−2^ is shown in Figure S28 (Supporting Information). In agreement with the previously discussed results, cells cycled in the DZS electrolyte demonstrate exceptional stability, maintaining a CE of 99.9% over 5300 cycles. In contrast, the cell using the ZS electrolyte exhibits severe CE fluctuations after only 22 cycles, likely due to the onset of uncontrolled dendrite formation.

Figures 5d and S29a,b (Supporting Information) further evaluate CE under more demanding conditions, 10 mA cm^−2^ and a fixed areal capacity of 1 mAh cm^−2^. In the ZS electrolyte, the CE rapidly declines within just a few cycles, indicating early‐stage internal short‐circuiting. Remarkably, the inclusion of Dy^3+^ in the DZS electrolyte enables highly stable cycling for up to 7000 cycles while preserving a CE of 99.8%.

Even under extreme conditions, 87.1% DOD, 60 mA cm^−2^ current density, and 15 mAh cm^−2^ areal capacity (Figure 5e), the Zn||Cu cell with DZS electrolyte sustains a CE above 99% for over 100 cycles. In stark contrast, the ZS‐based counterpart begins with a much lower initial CE of 81.9%, which quickly drops to 68.3% within only 5 cycles. When benchmarked against state‐of‐the‐art Zn‐based systems (Figure 5f; Table S3, Supporting Information), the Zn||Cu cell utilizing the DZS electrolyte demonstrates markedly enhanced cycling stability and Zn reversibility, with record CPC values.

To evaluate the performance of the DZS electrolyte in a full‐cell configuration, AZIBs were assembled using a Zn metal anode and a V_2_O_5_ cathode. The V_2_O_5_ cathode was synthesized via a hydrothermal method and exhibited a characteristic flake‐like morphology (Figures S30 and S31a,b, Supporting Information). CV profiles (Figure S32, Supporting Information) revealed negligible differences between the two electrolytes, indicating that Dy^3+^ does not significantly affect the intrinsic redox behavior of the V_2_O_5_ cathode.^[^ ^22^ ^]^

Cycling performance and corresponding GCD profiles at 1 A g^−1^ are shown in Figures S33–S35 (Supporting Information). In the ZS electrolyte, the full cell failed after only 39 cycles due to short‐circuiting caused by Zn dendrite penetration. In stark contrast, the DZS‐based cell delivered a high specific capacity of 410.7 mAh g^−1^ with excellent capacity retention of 99.1% after 200 cycles.

To further assess long‐term durability, high‐rate cycling was performed at 10 A g^−1^ (Figure S36, Supporting Information). The V_2_O_5_||Zn cell with DZS electrolyte retained 80% of its initial capacity over 5000 cycles with ≈100% CE, whereas the ZS‐based counterpart exhibited continuous capacity fading due to dendrite‐induced degradation.

To assess practical applicability, V_2_O_5_||Zn pouch cells incorporating the DZS electrolyte and a low negative‐to‐positive (N/P) capacity ratio of 1.18 were fabricated and tested (Figure S37, Supporting Information). The DZS‐based cell retained 83.3% of its capacity at 1 mA cm^−2^, while the equivalent cell with ZS electrolyte experienced rapid capacity loss. Postmortem analysis of the disassembled pouch cell revealed markedly inhomogeneous Zn deposition in the ZS electrolyte (Figure S38, Supporting Information), with platelet‐like protrusions extending from the anode surface and posing a risk of separator penetration and mechanical damage. In contrast, the Zn anode cycled in DZS exhibited a compact, uniform morphology, indicating effective dendrite suppression. Finally, Figure S39 (Supporting Information) shows how a pouch‐type cell successfully powers a light‐emitting diode (LED) display, demonstrating the potential of the DZS electrolyte for practical and scalable AZIB energy storage applications.

## 3 Conclusion

In this study, we presented a rational electrolyte‐engineering strategy to regulate Zn crystal growth in AZIBs. By introducing DyCl_3_ into a baseline ZS electrolyte, we effectively modified the conventional 2D epitaxial growth along the [$10\bar{1}0$] direction and instead promoted a more homogeneous Zn growth resembling a screw dislocation‐driven growth mechanism along the [0001] orientation. This transition leads to the formation of dense, compact, and dendrite‐free Zn plating. As a result, Zn||Zn symmetric cells exhibit outstanding long‐term cycling stability, operating for over 8500 h at 1 mA cm^−2^ and 3000 h at 10 mA cm^−2^. Remarkably, even under a high DOD of 87.1%, stable cycling is sustained for 150 h at an ultrahigh current density of 60 mA cm^−2^. Full cells assembled with V_2_O_5_ cathodes and Zn anodes under limited Zn inventory conditions (N/P = 1.18) demonstrate excellent capacity retention and rate capability. This work underscores the critical importance of facet‐selective electrolyte modulation in controlling metal plating pathways. Beyond advancing fundamental understanding of crystal growth in metal anodes, it provides a crystal‐growth‐informed, scalable, and effective framework for designing dendrite‐free, high‐performance AZIB systems. These insights are expected to guide the future development of electrolyte additives and electrode architectures tailored for next‐generation aqueous energy storage technologies.

## Conflict of Interest

The authors declare no conflict of interest.

## Supporting information



Supporting Information

## Data Availability

The data that support the findings of this study are available from the corresponding author upon reasonable request.
