# Supplementary material for: Crystal Growth Engineering for Dendrite‐Free Zinc Metal Plating
Source: Adv Mater. 2025 Sep 30;38(51):e10906. doi: 10.1002/adma.202510906 (PMC13568996; doi:10.1002/adma.202510906)
Supplement: Supplementary file 1 [file ADMA-38-e10906-s001.pdf]

Experimental Section

***Chemicals*:** All chemicals were used without further purification. Dysprosium(III) chloride ($DyCl_3$) was purchased from Thermo Scientific. Zinc sulfate heptahydrate ($ZnSO_4·7H_2O$), Zn foil, Cu foil, and carbon cloth were bought from Canrd Technology.

***Preparation of the Electrolytes*:** The base 2 M $ZnSO_4$ electrolyte (ZS) was prepared by dissolving $ZnSO_4·7H_2O$ into 100 mL deionized (DI) water. The Dy^{3+}-modified electrolyte (DZS) was prepared by adding a 0.05 M concentration of $DyCl_3$ into the ZS electrolyte.

***Materials Characterizations*:** An Auriga Zeiss field emission scanning electron microscope (SEM) was used to characterize the morphology of Zn foil and V_2O_5 materials. The X-ray diffraction (XRD) patterns were obtained using a Bruker AXS D8 diffractometer. A Bruker Alpha FTIR spectrometer and the Renishaw Invia Reflex Raman spectra (532 nm) were used to determine the solvation structure of the electrolyte. The 3D morphology evolutions of deposited Zn were recorded by a Sensofar confocal microscope.

***Simulation methods*:** First-principles calculations were performed using the DMol3 module. The generalized gradient approximation (GGA) and Becke–Lee–Yang–Parr (BLYP) functionals were employed to describe electron interaction energies. Ultrasoft pseudopotential was performed (OTFG describing the valence electrons) with Grimme's method introduced for DFT-D correction. The self-consistent field (SCF) tolerance was set to $5×10^{-7}$ eV $atom^{-1}$, with a cutoff energy of 500 eV and a maximum of 500 cycles. Additionally, a vacuum space of 20 Å was included in each simulation model. Adsorption energies (∆E) were calculated using the following equation: $\Delta E = E_{total} - E_{atom} - E_{mol}$ (E_{total}, the total energy of the system; E_{atom}, the total energy of the matrix; and E_{mol}, the total energy of Zn^{2+}). In terms of the diffusion energy barrier calculations, transition state (TS) search tasks were implemented using the DMol3 module. Geometry optimizations were conducted prior to the TS search simulations.

MD simulations were conducted using the Forcite module. The forcefield was set to be COMPASSIII, and the electrostatic employed the Ewald electrostatic method with the van der Waals method assigned as atom-based. The aqueous electrolyte was modeled by defining 2,000 H_2O molecules, 76 Zn^{2+}, and 76 SO_4^{2-}, corresponding to a 2 M $ZnSO_4$ aqueous solution. The forcefield types were assigned according to the states of the atoms. Since the forcefield for Dy is not available in the Forcite database, La atoms and the corresponding La-based forcefield were employed instead, given that Dy belongs to the lanthanide series. Geometry optimizations were conducted prior to the MD simulations. To enhance the efficiency of the

MD simulations, parallel calculations were performed. Both first-principles and MD simulations were performed based on the Materials Studio platform.

The finite element analyses of Zn plating were conducted using ANYSES based on the Nernst–Planck–Poisson equation. In terms of the electrochemistry on the Zn anode surface, the concentration-dependent Butler–Volmer equation was introduced. Semiellipse nuclei were set at the initial state at the Zn anode surface. In terms of the phase-field simulations, the modeling domain is set to be 3 × 6 μm^2, where the solid dendritic phase grows from the bottom boundary and the top boundary is set to be the electrolyte. The solid phase is set to be pure Zn metal with an electric conductivity of 1.7×10^7 S m^{-1}, while the Zn^{2+} diffusion coefficient in the Zn metal electrode is set to 3.7×10^{-13} m^2 s^{-1}. The electrolytes were set following the compositions of ZS and DSZ. The surface energy (κ) is constant with a value of 5×10^{-5} J m^{-1}. According to Zn hexagonal crystal structure, the mode of the anisotropy is set to ω = 6. Three defect positions are set on the Zn electrode plane to facilitate dendrite nucleation. According to the different modification conditions of the surface, the growth morphology of dendrites is adjusted by adjusting the anisotropic intensity (α) from 0.1 to 0.2.

***Electrochemistry*:** The Zn||Zn, Zn||Cu, and Zn||V_2O_5 cells were assembled by CR2032 coins using glass fiber (GF-B) as the separator. The cathode was prepared using 70% V_2O_5 active materials, 20% Ketjenblack, and 10% polyvinylidene fluoride (PVDF). Each cell was filled with 100 μL electrolyte. Cyclic voltametry (CV) and linear sweep voltametry (LSV) measurements were tested on a Correst electrochemical workstation. Galvanostatic charge/discharge (GCD) tests were measured on the Neware battery test system (Shenzhen, China).
The corrosion current density was determined by the Tafel extrapolation method in the high-polarization region. Briefly, the Tafel relation, the high-overpotential limit of Butler–Volmer, can be written as:

$$\eta = E - E_c = a \pm b \log_{10} |i|$$

$$\eta = \pm b \log_{10} \left(\frac{i}{i_c}\right)$$

which is valid at relatively high overpotentials ($|\eta| \geqslant 50$ mV, depending on the system). In this regime, and under charge-transfer control (fast mass transport), each branch (anodic/cathodic) reflects a single reaction and is quasi-linear on a semi-log plot. Excessively large overpotentials can activate vigorous hydrogen evolution and other secondary effects (bubble coverage, local pH shifts, mass-transport limits, iR drop, surface changes) that distort intrinsic kinetics and degrade the extrapolation. For all datasets, we used the same overpotential window (60–120 mV) for the linear Tafel fits. Practically, i_c was obtained by fitting the linear anodic and cathodic segments and extrapolating them back to $\eta = 0$ ($E = E_c$, coincident with

the steady-state open circuit voltage). The intersection of these lines yields i_{corr} and E_{corr}. Figure S8 schematizes the procedure.

To substantiate the extracted i_c and E_c, we independently estimated icorr using the Stern–Geary (linear polarization resistance) method to cross-check:[1]

$$i_c = B/R_p$$

$$B = \frac{\beta_a \beta_c}{2.303(\beta_a+\beta_c)}$$

$$R_p = \frac{dE}{di}$$

where R_p is obtained from a small-polarization scan near E_c, and β_a, β_c are the Tafel coefficients of the anodic and cathodic reactions.

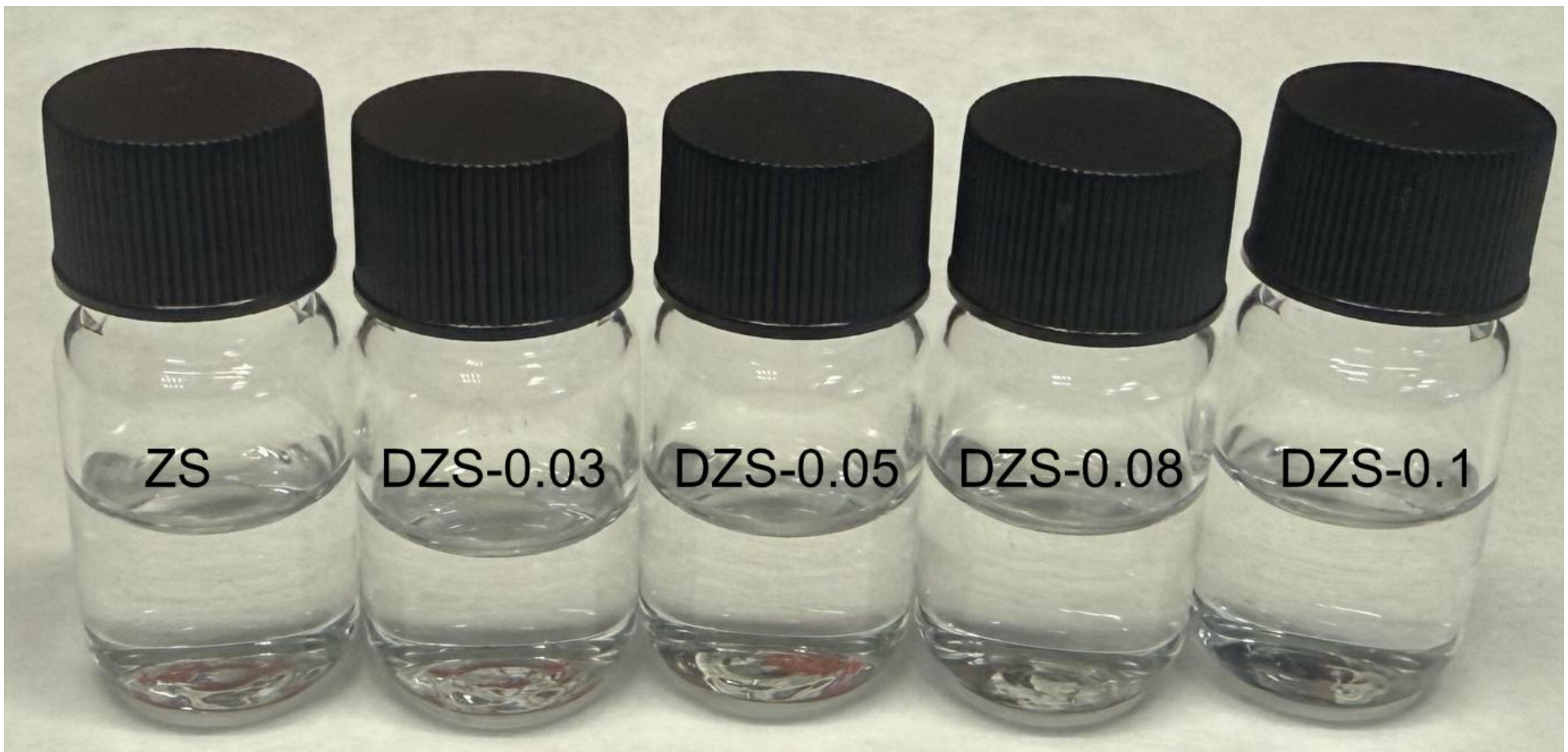

Figure S1. Photographs of the ZS and DZS electrolytes with different concentrations of $DyCl_3$ additive.

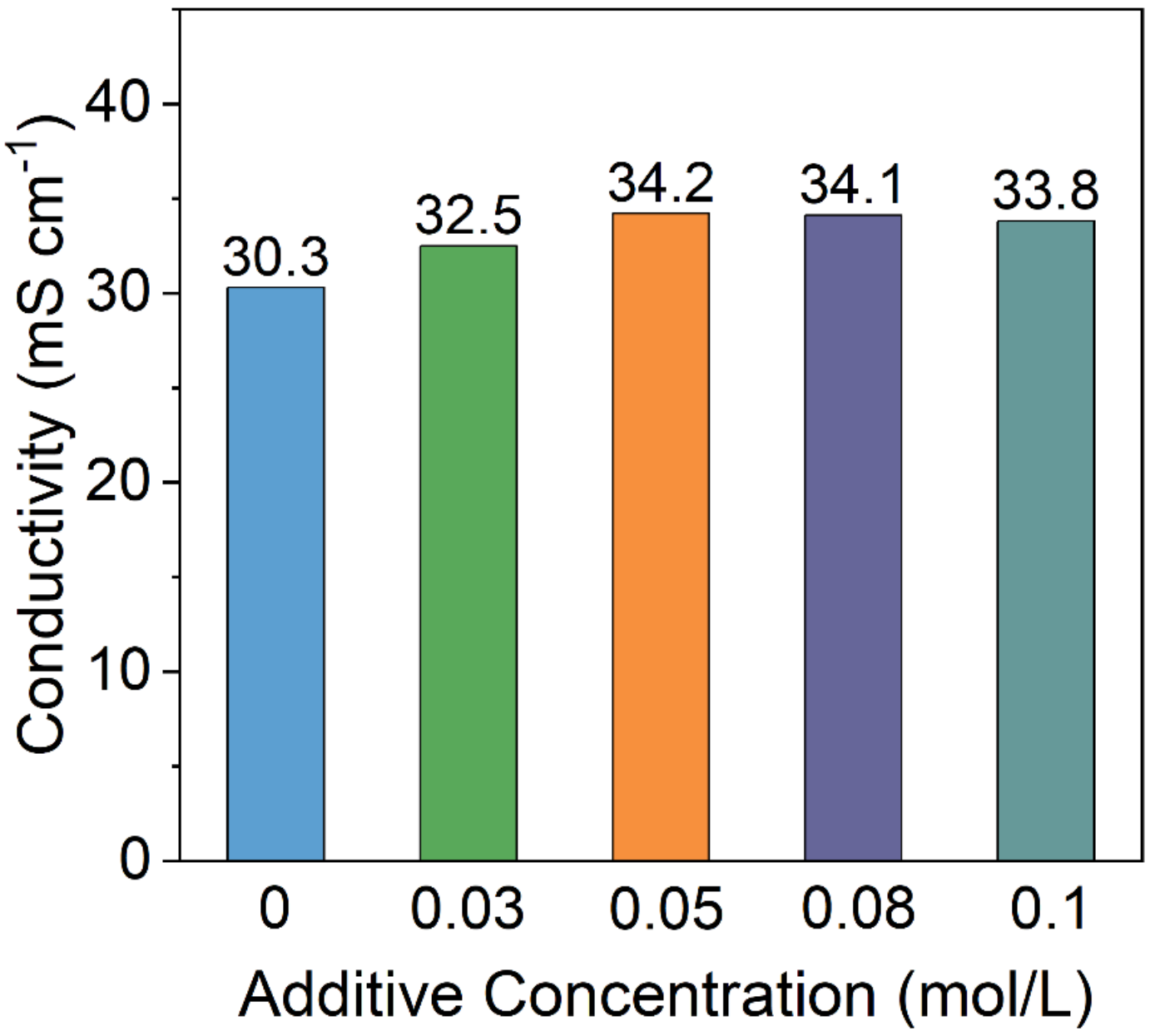

Figure S2. Ionic conductivities of ZS and DZS electrolytes.

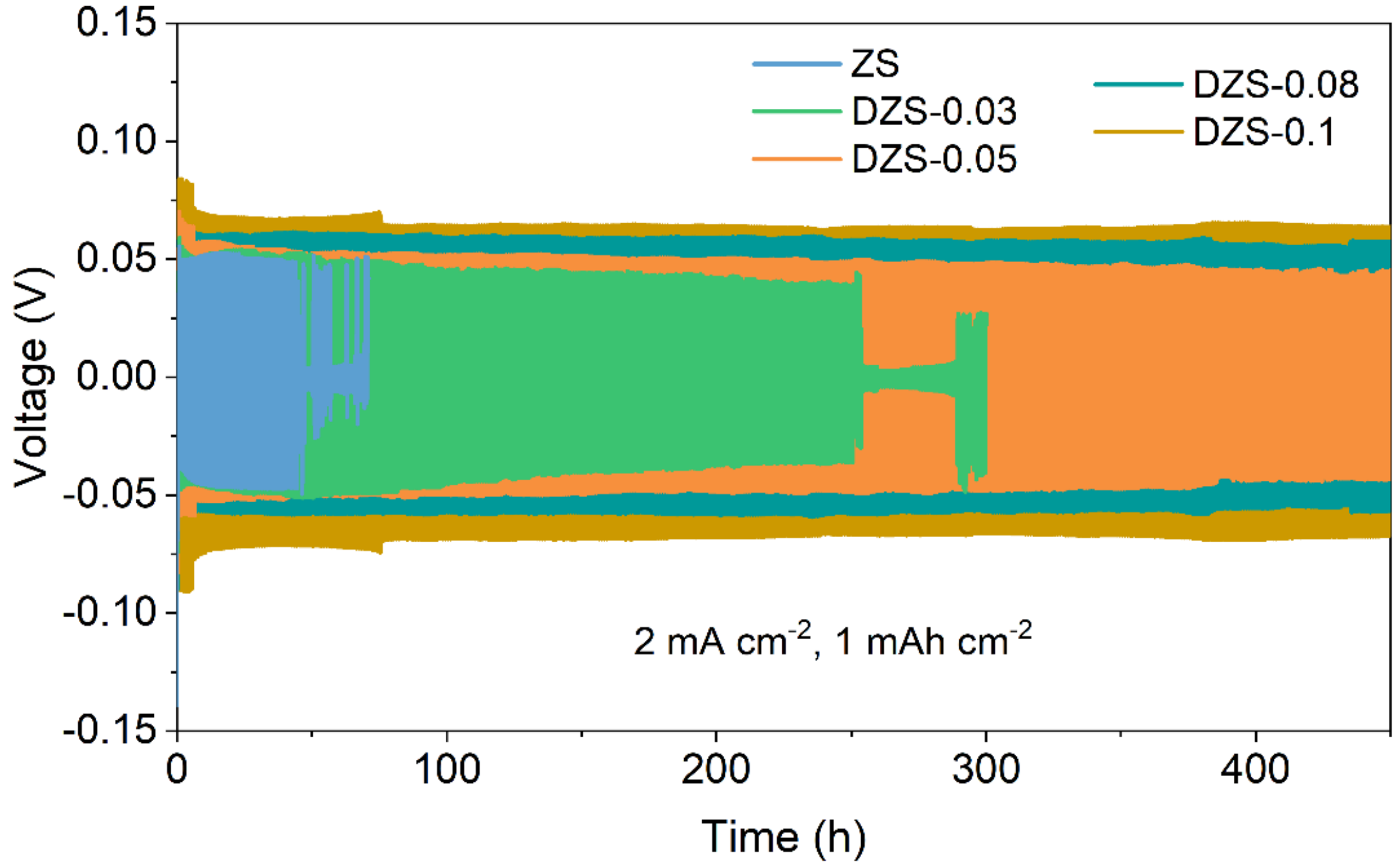

Figure S3. Electrochemical performance of Zn||Zn cells tested in ZS electrolyte with different concentrations of $DyCl_3$.

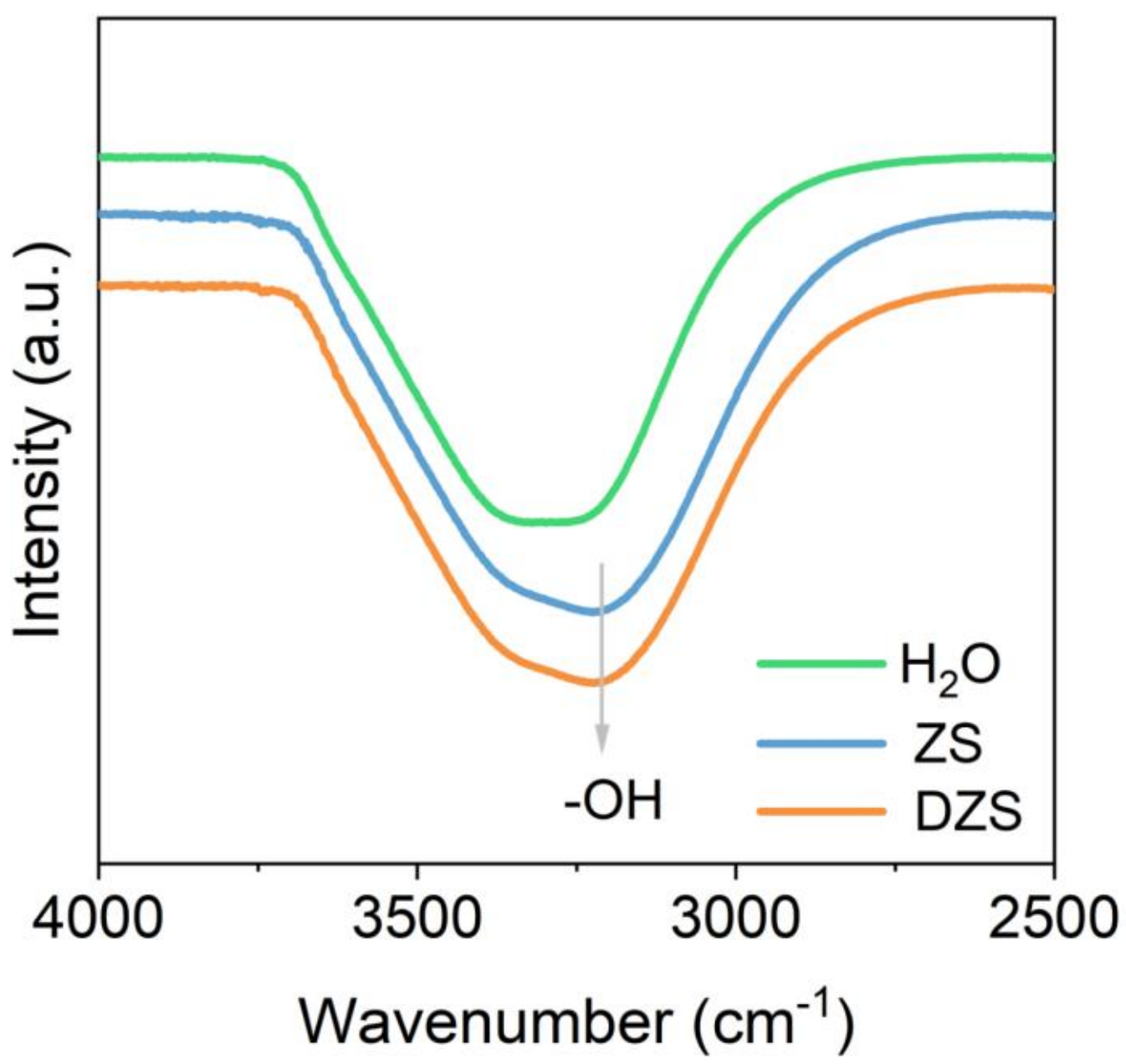

Figure S4. FTIR spectra of ZS and DZS electrolyte.

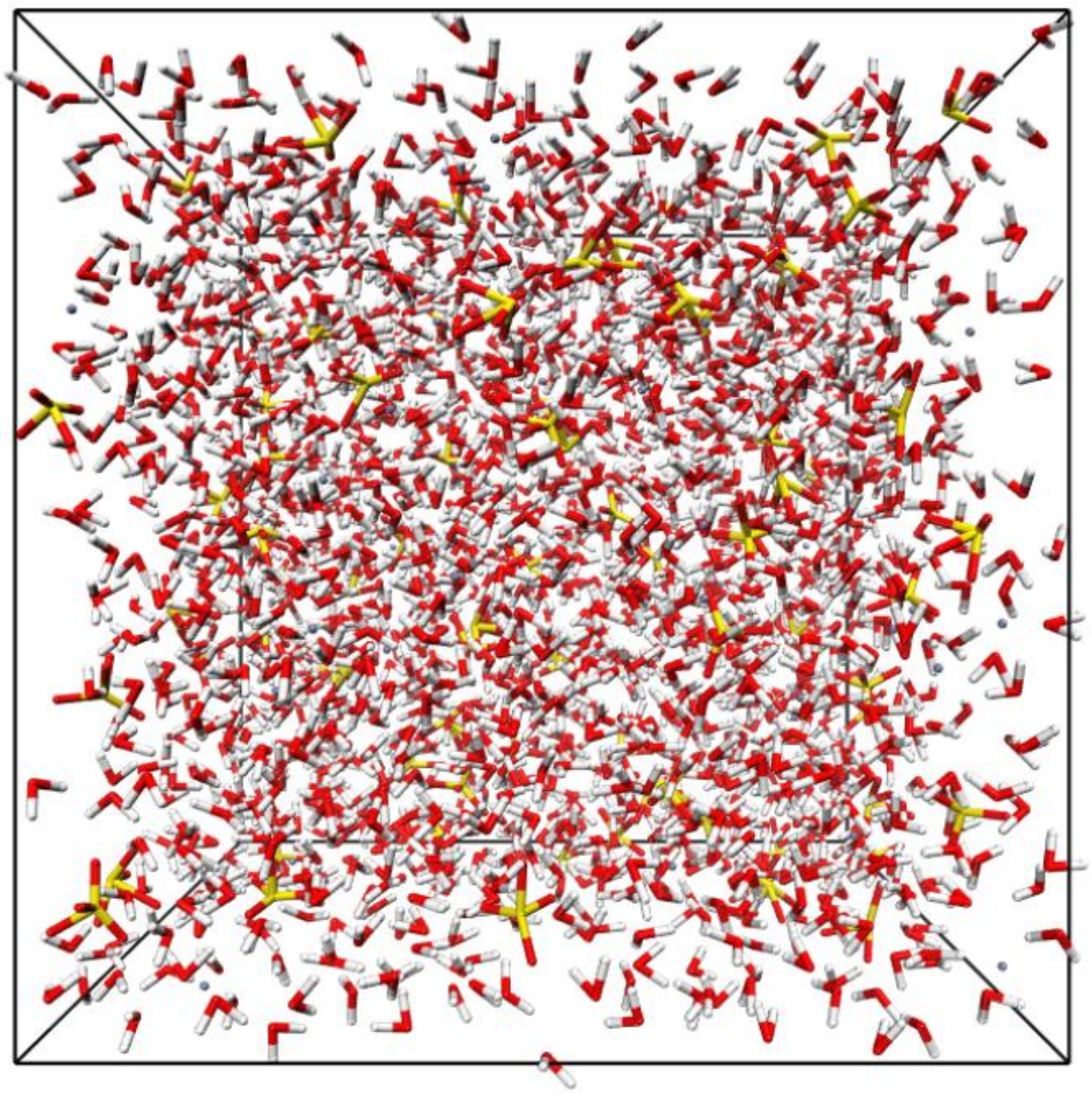

Figure S5. Structure of the ZS electrolyte used for MD simulation.

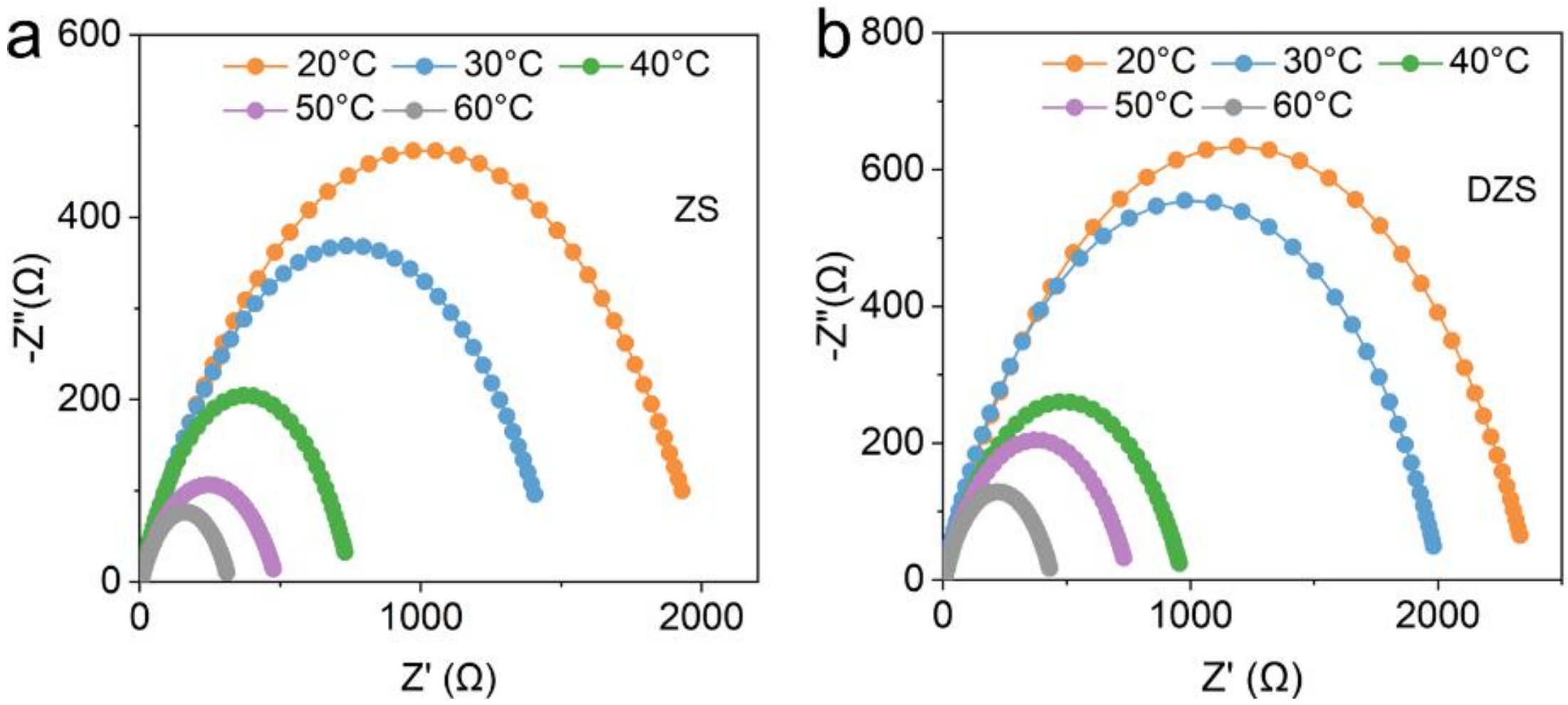

Figure S6. EIS spectra of ZS (a) and DZS (b) electrolytes at different temperatures. (c) Arrhenius plots and corresponding activation energies.

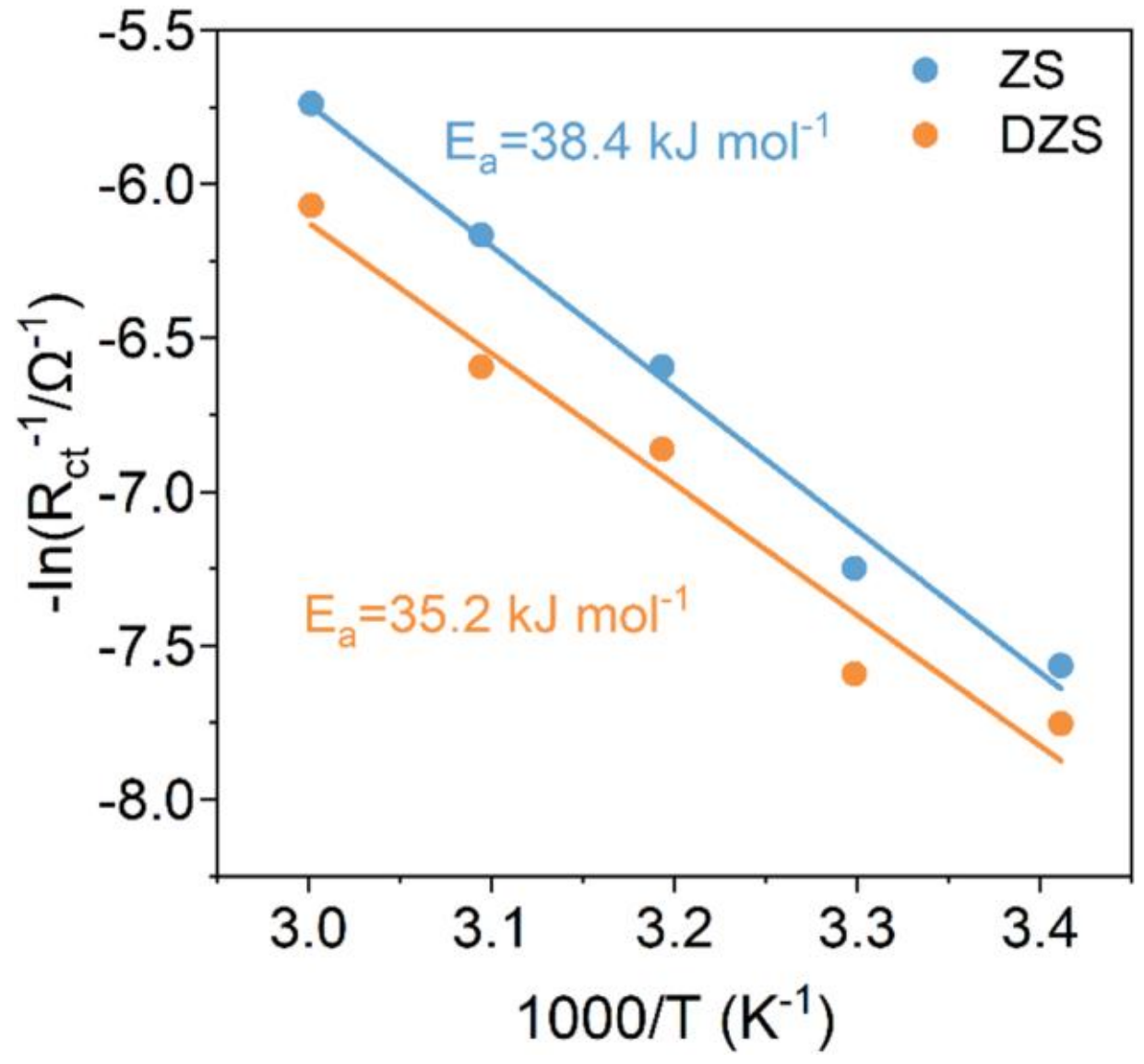

Figure S7. Arrhenius plots of the charge transfer resistance and corresponding activation energies for ZS and DZS electrolytes.

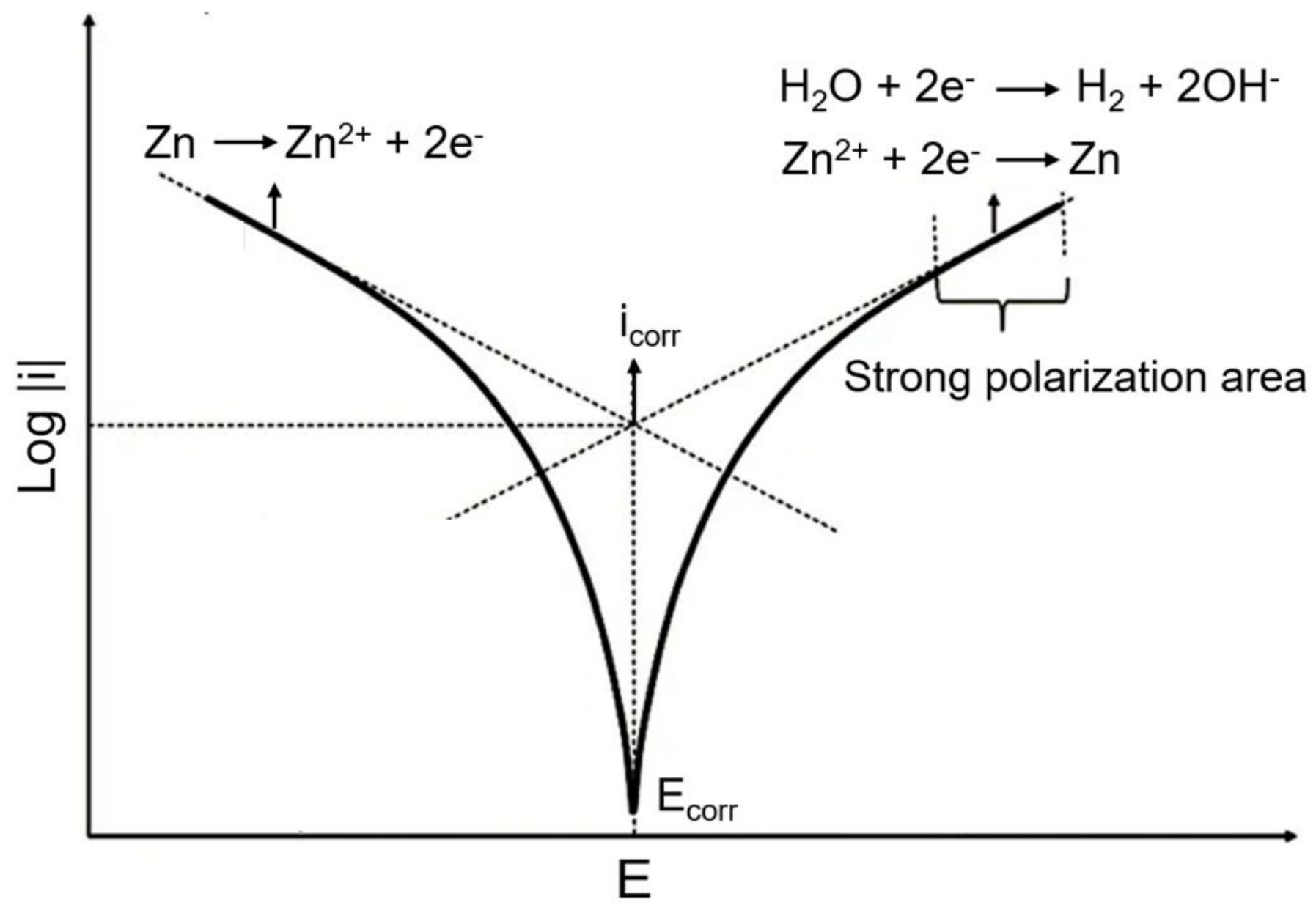

Figure S8. Schematic diagram of the method used to determine the corrosion potential and current.

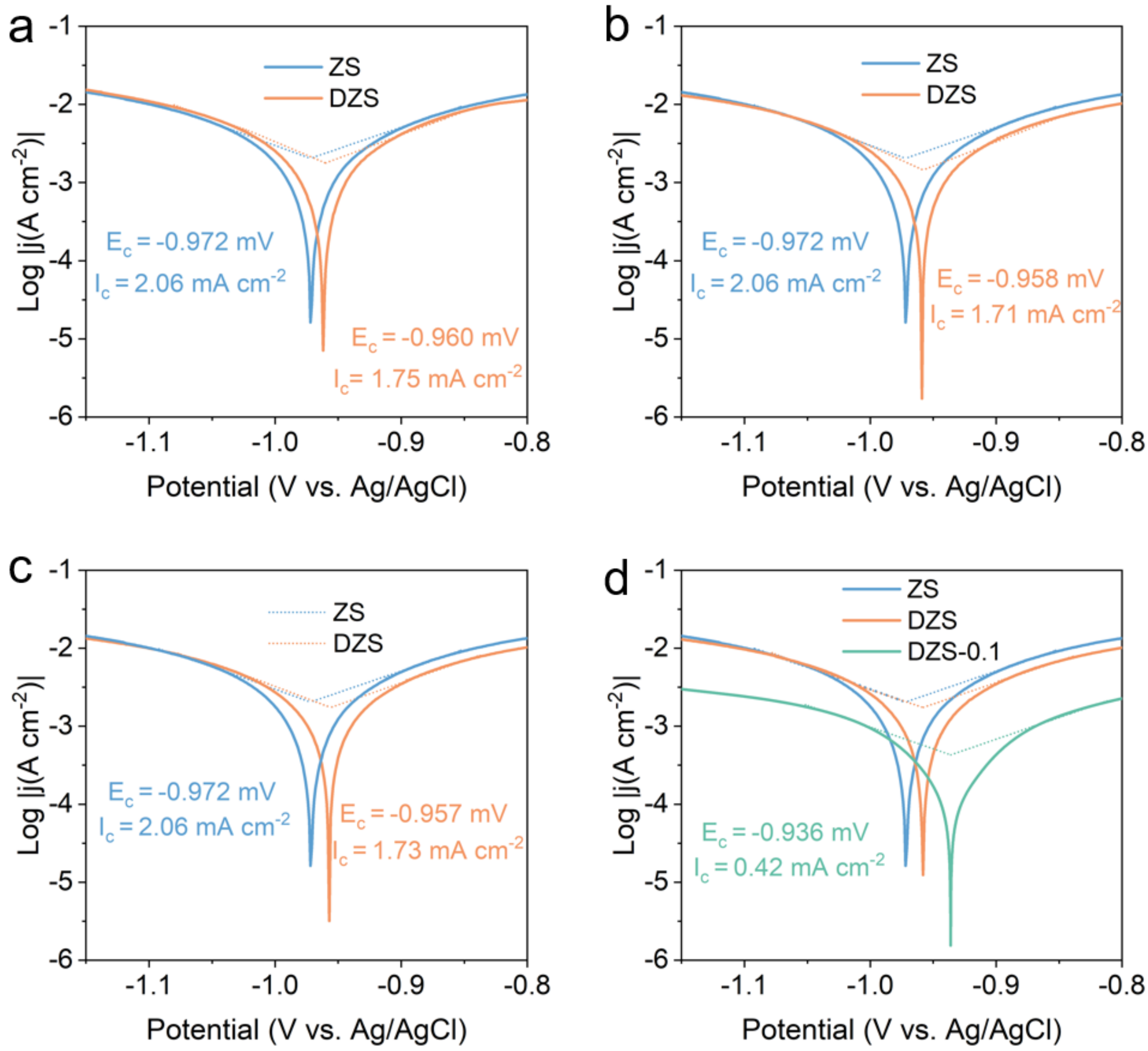

Figure S9. (a-c) Three sets of Tafel plots for ZS and DZS. d) Tafel plots for DZS-0.1.

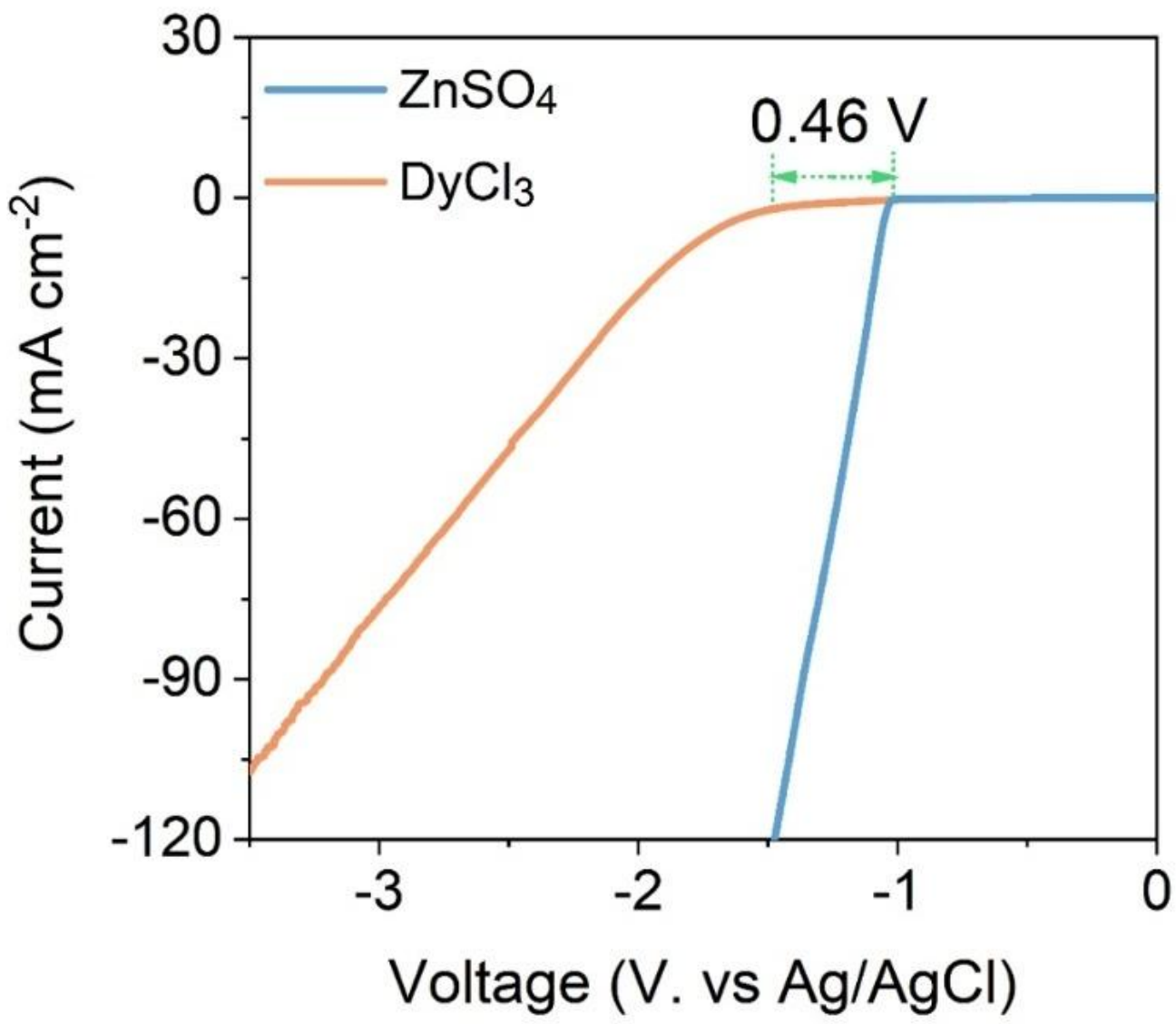

Figure S10. LSV curves of $ZnSO_4$ and $DyCl_3$ electrolytes at the scan rate of 1 mV s^{-1}.

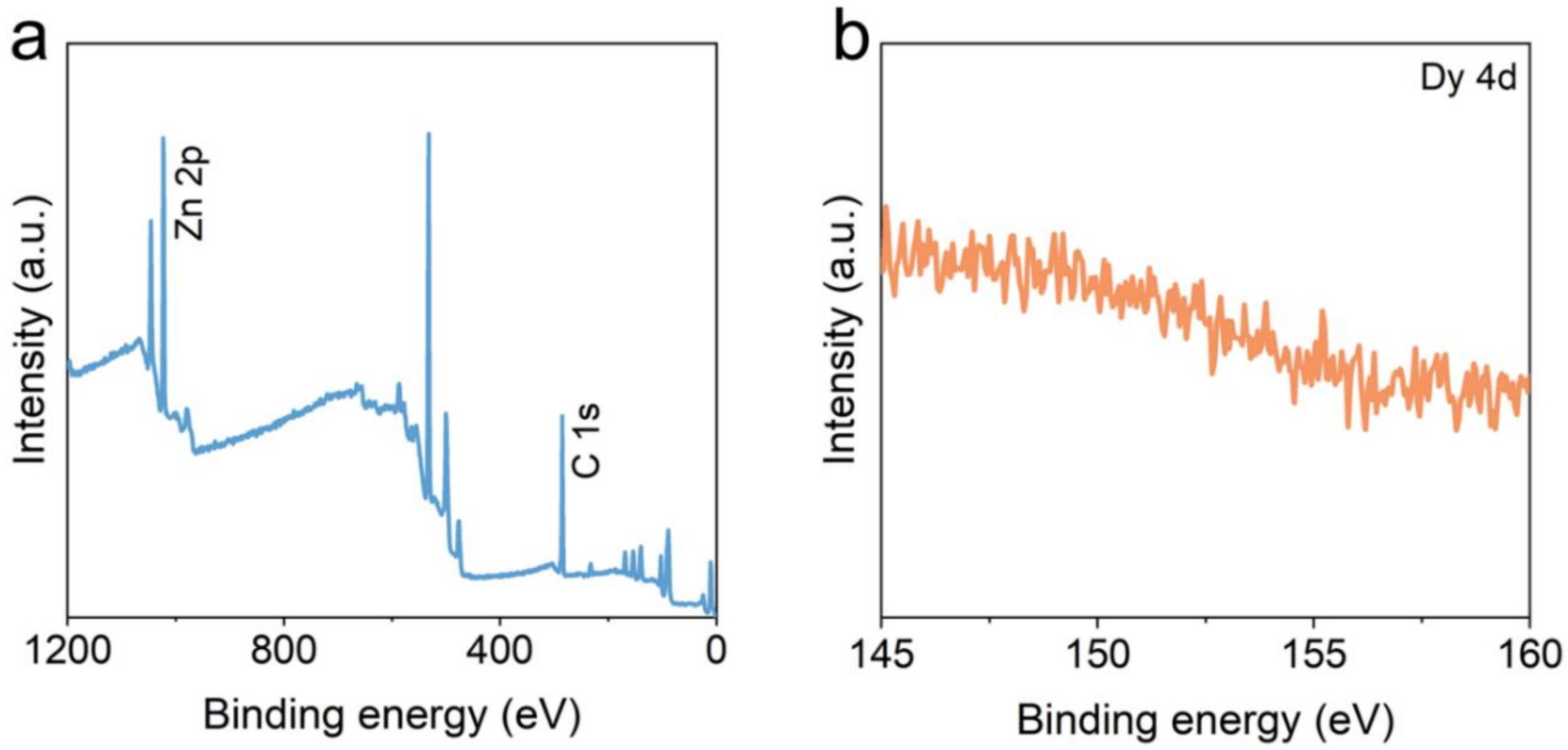

Figure S11. Survey (a) and high-resolution Dy 4d (b) XPS spectra.

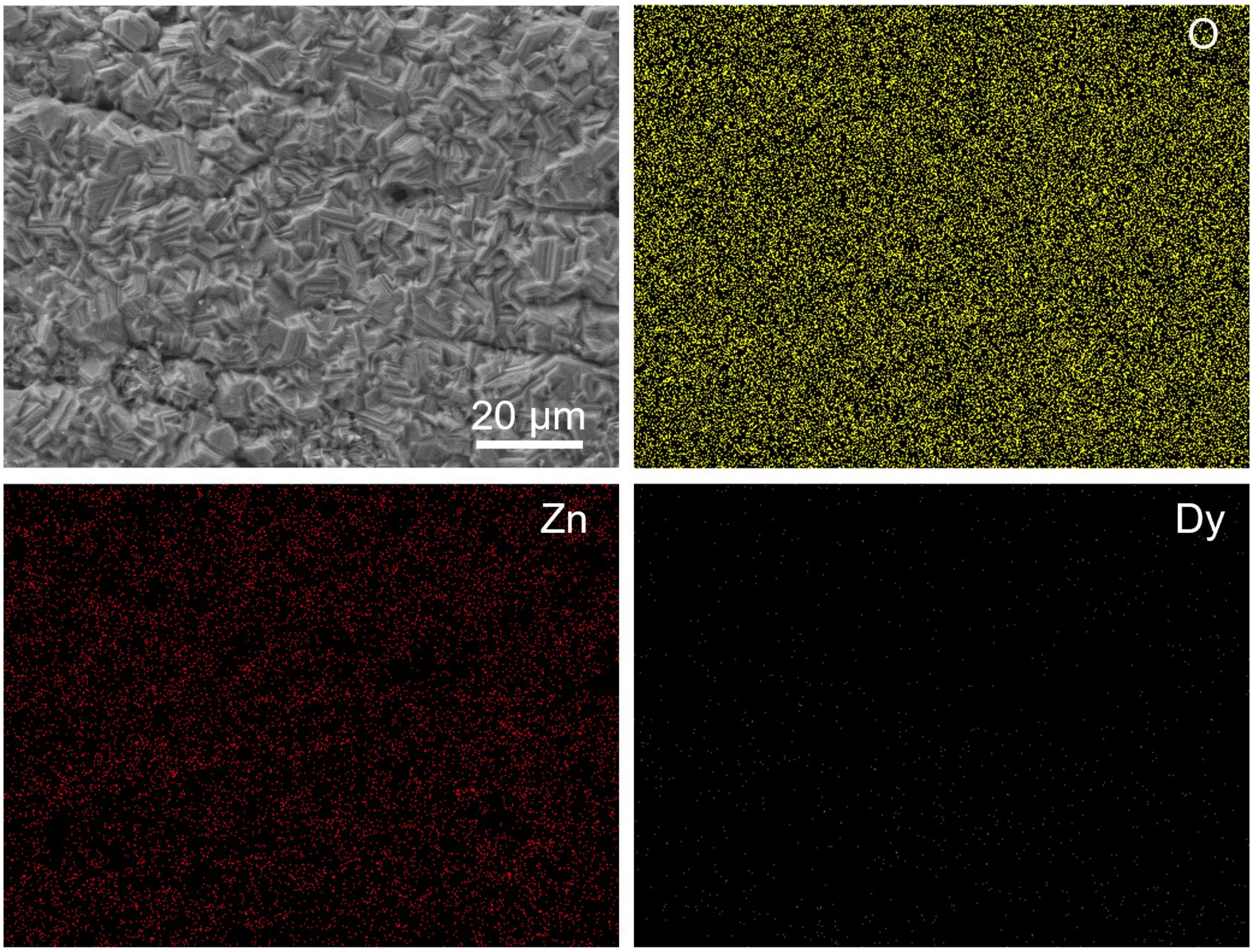

Figure S12. SEM image and EDS compositional maps of the cycled Zn anode in DZS.

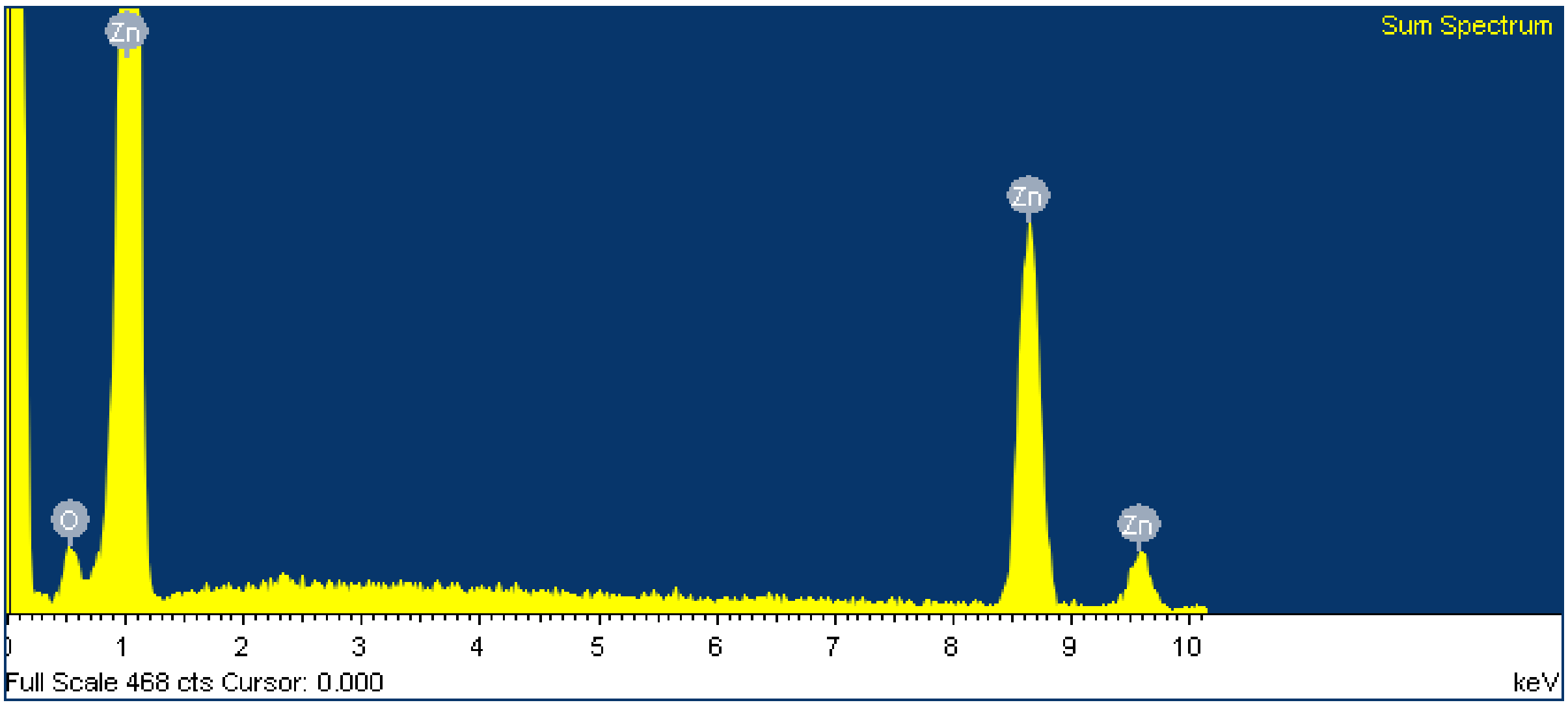

Figure S13. EDS profile of the cycled Zn anode in DZS.

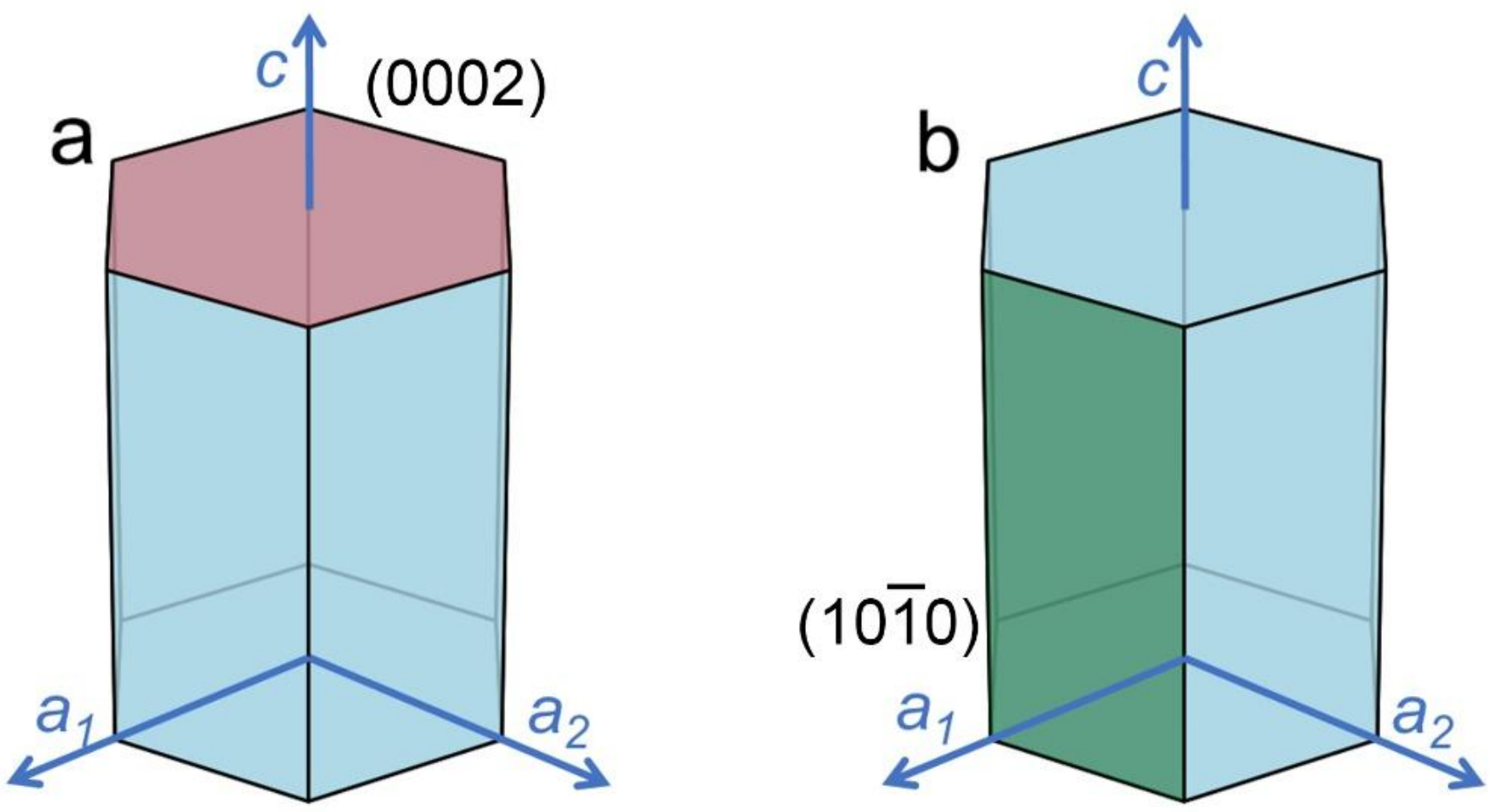

Figure S14. Schematic diagram of (0002) (a) and $(10\bar{1}0)$ (b) planes

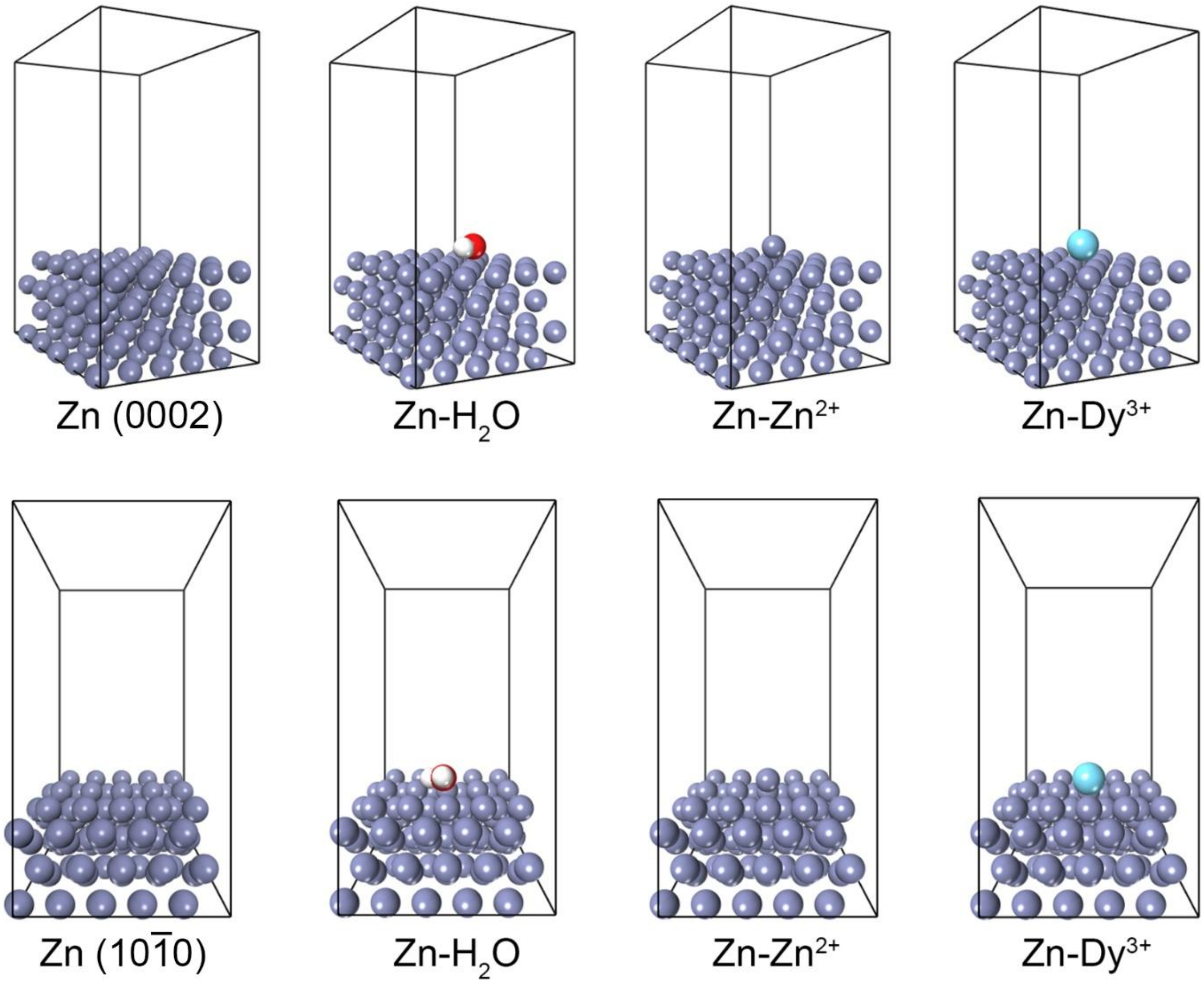

Figure S15. Initial models of (0002) and ($10\overline{1}0$) planes with various adsorption configurations.

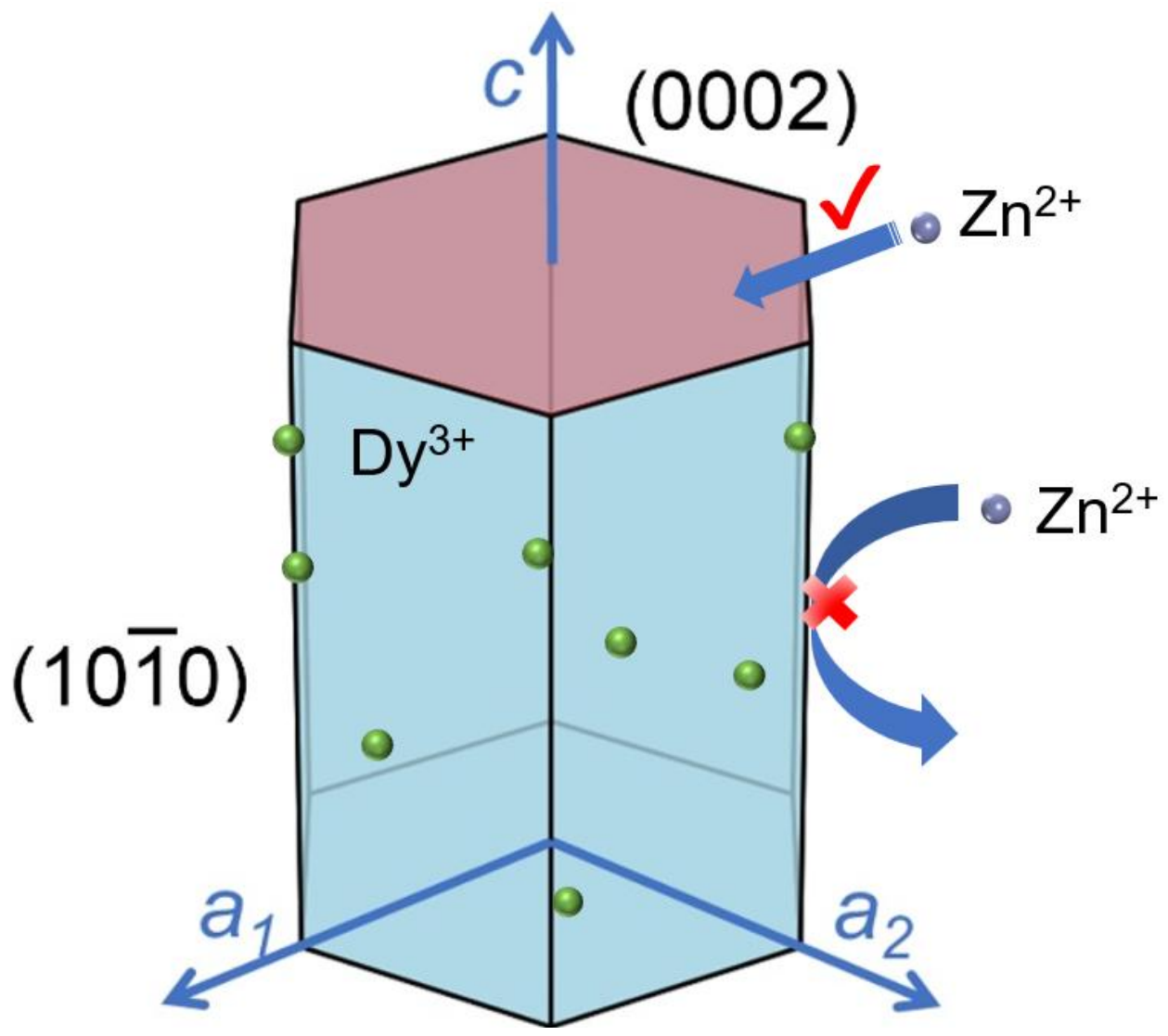

Figure S16. Schematic illustration of the selective adsorption of Dy^{3+} and its influence on the growth orientation of Zn metal.

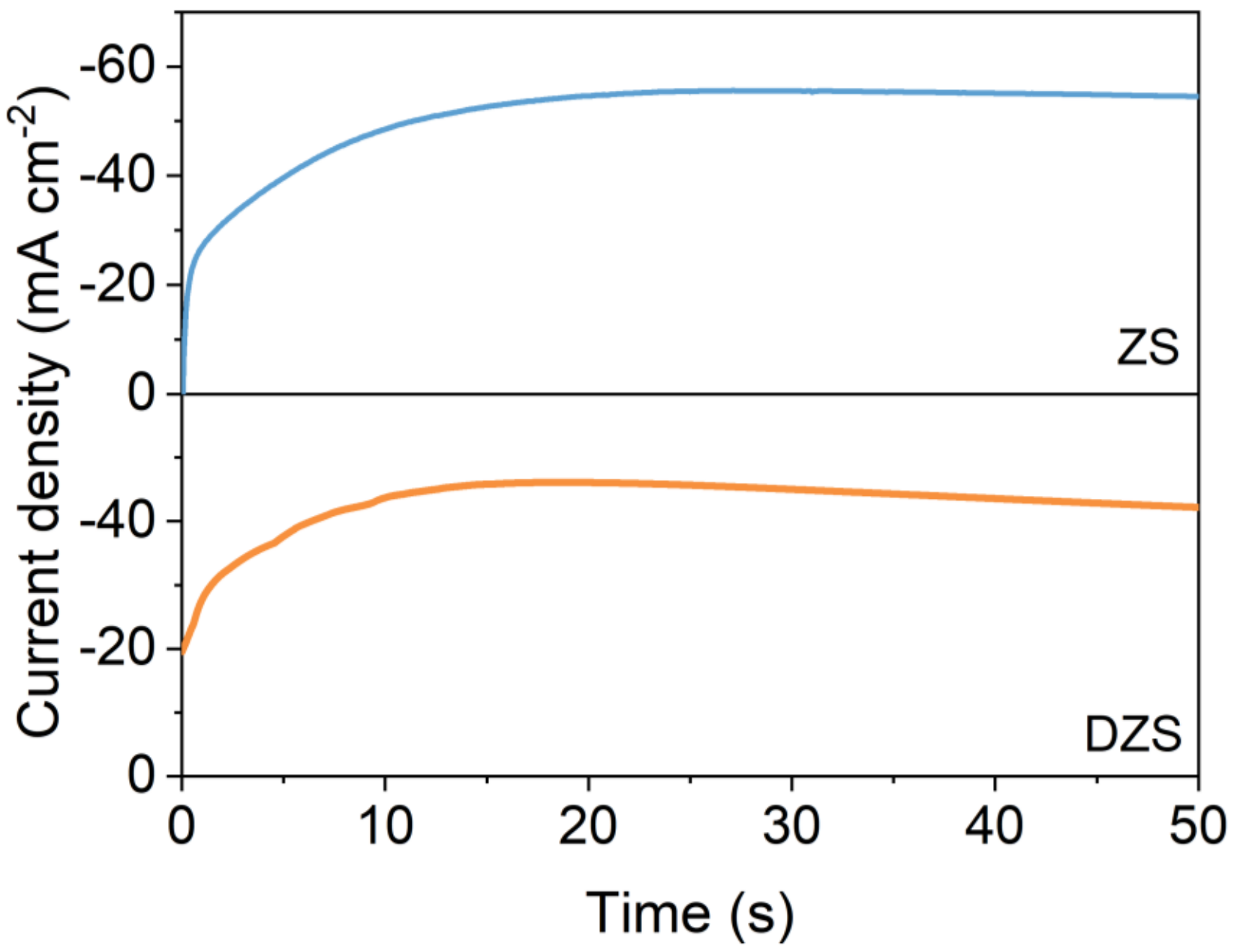

Figure S17. CA curves at -1.25 V.

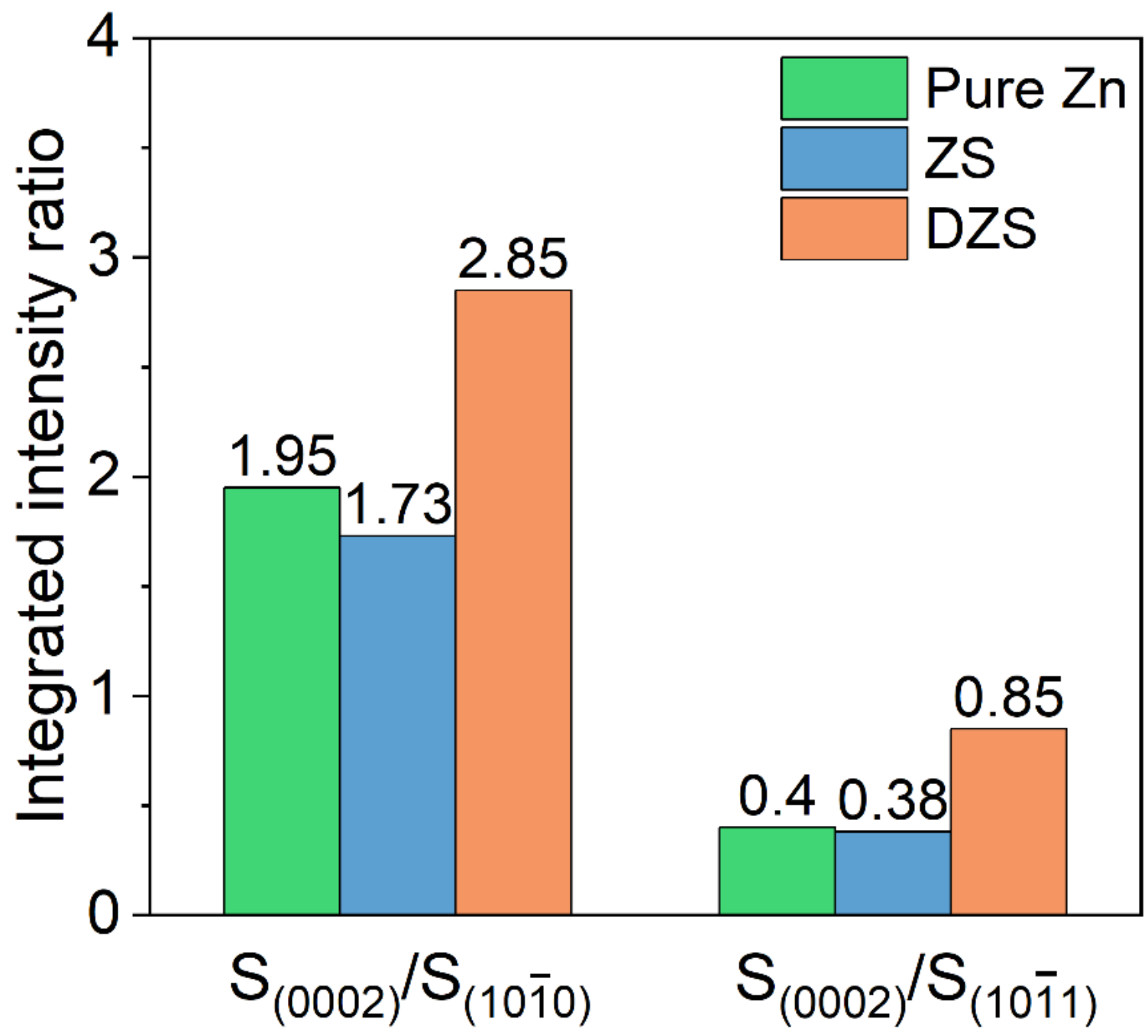

Figure S18. XRD peak integrated intensity ratios.

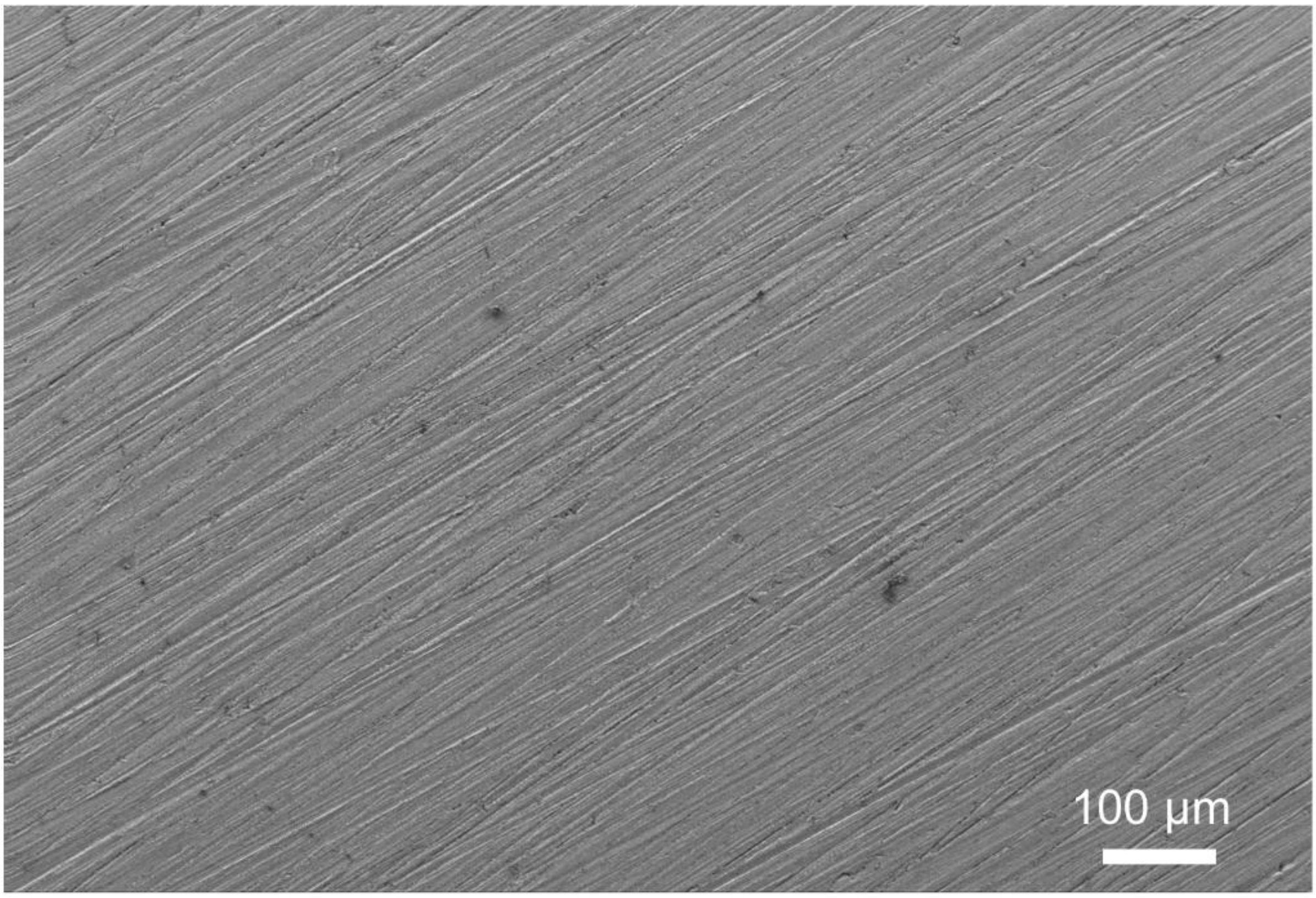

Figure S19. SEM image of the pristine polished Zn foil.

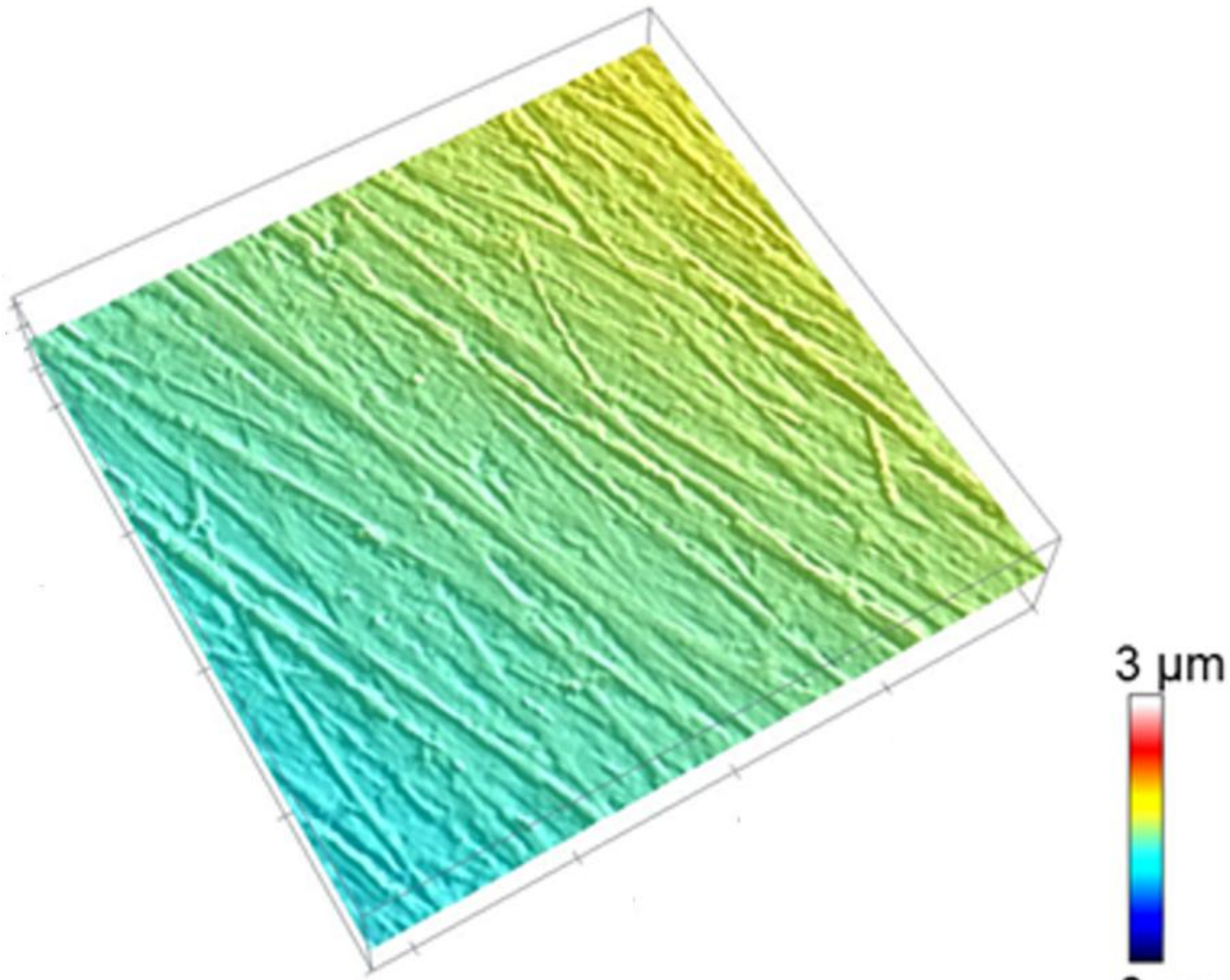

Figure S20. Confocal microscope 3D image of the Zn electrode surface before cycling.

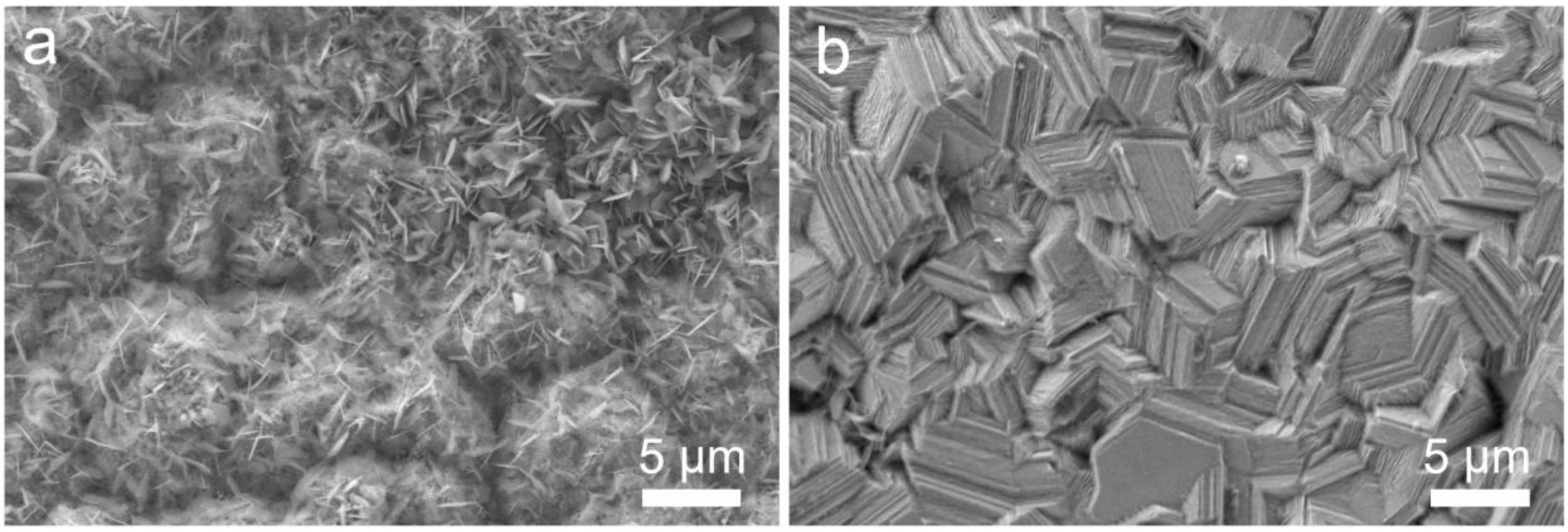

Figure S21. SEM images of the Zn plated onto a Cu substrate in ZS (a) and DZS (b) electrolytes, respectively.

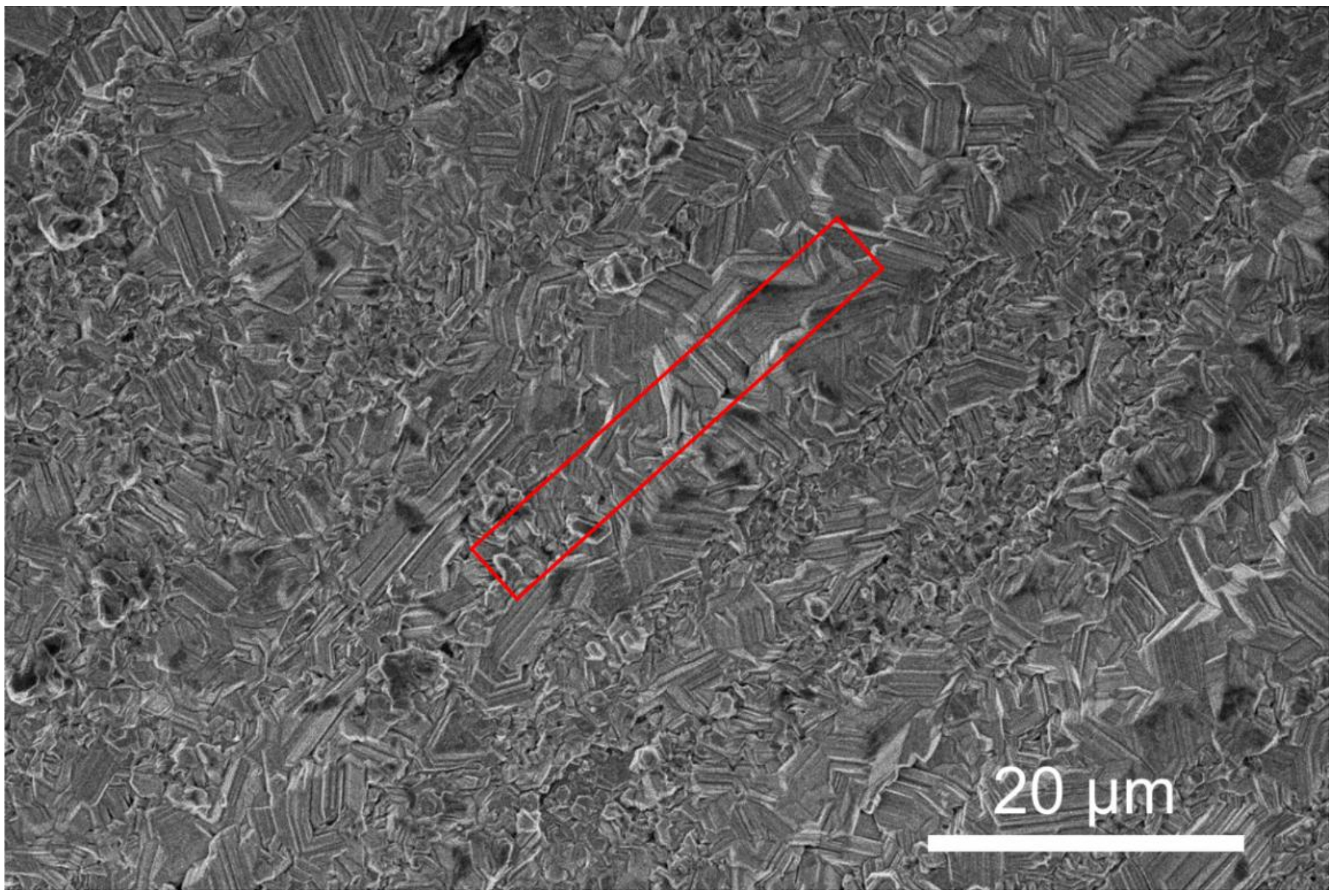

Figure S22. SEM image of plated Zn in DZS electrolyte. The sample used for cross-sectional analysis (Figure 4a) was prepared from the region marked in red.

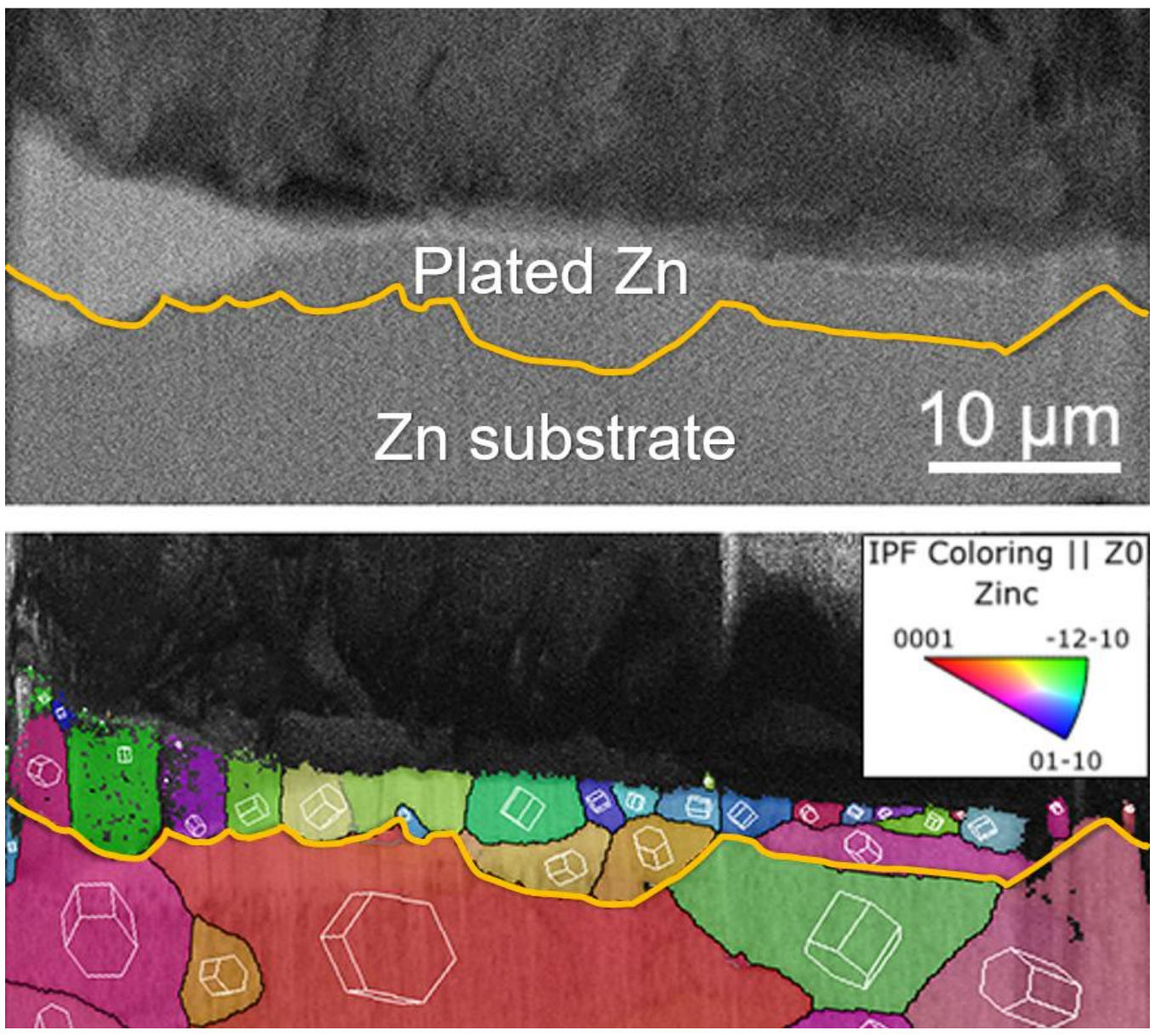

Figure S23. Cross-section SEM image and corresponding EBSD IPF of Zn plated in DZS electrolyte.

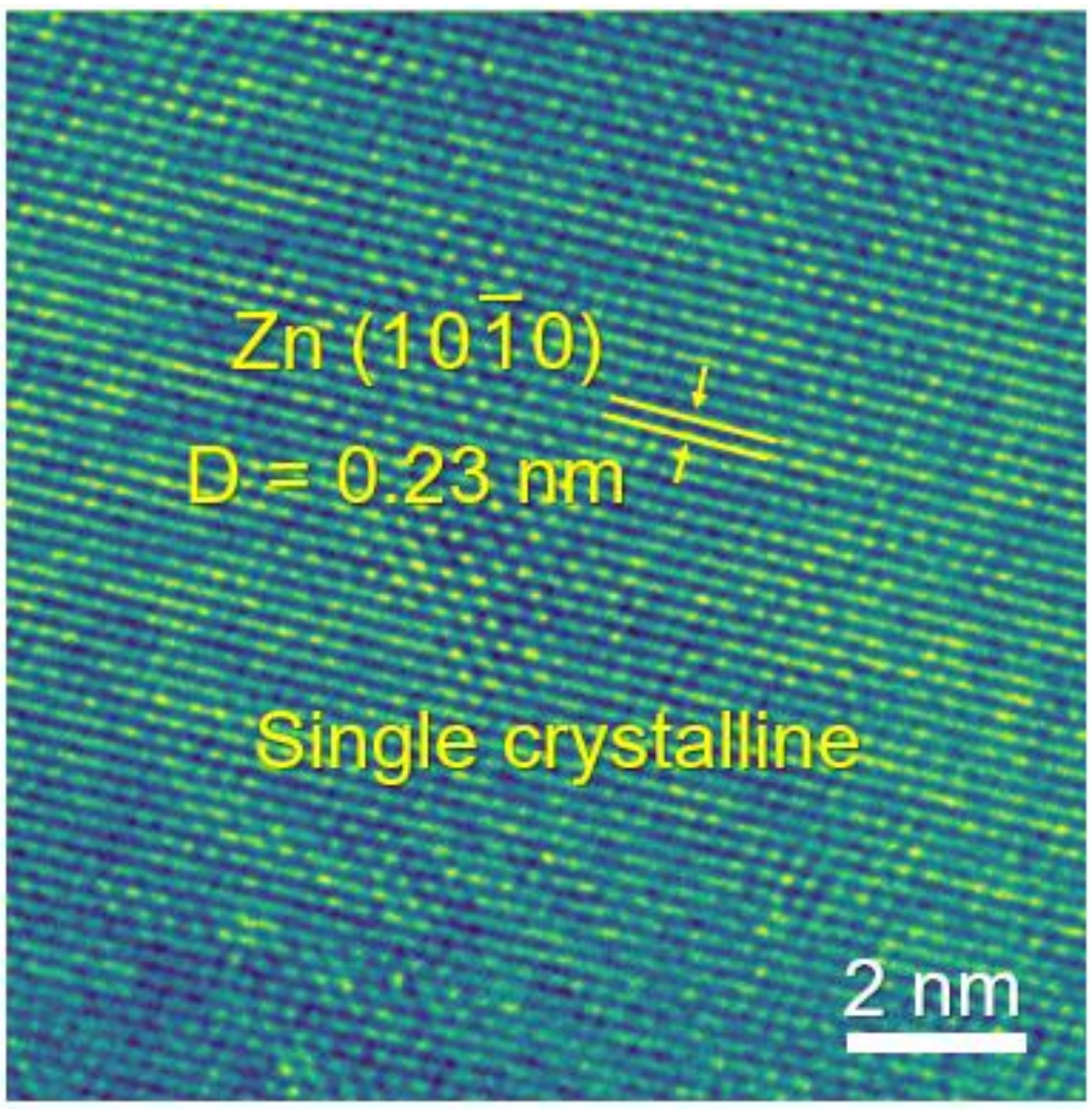

Figure S24. HRTEM image of a Zn grain in a plated layer.

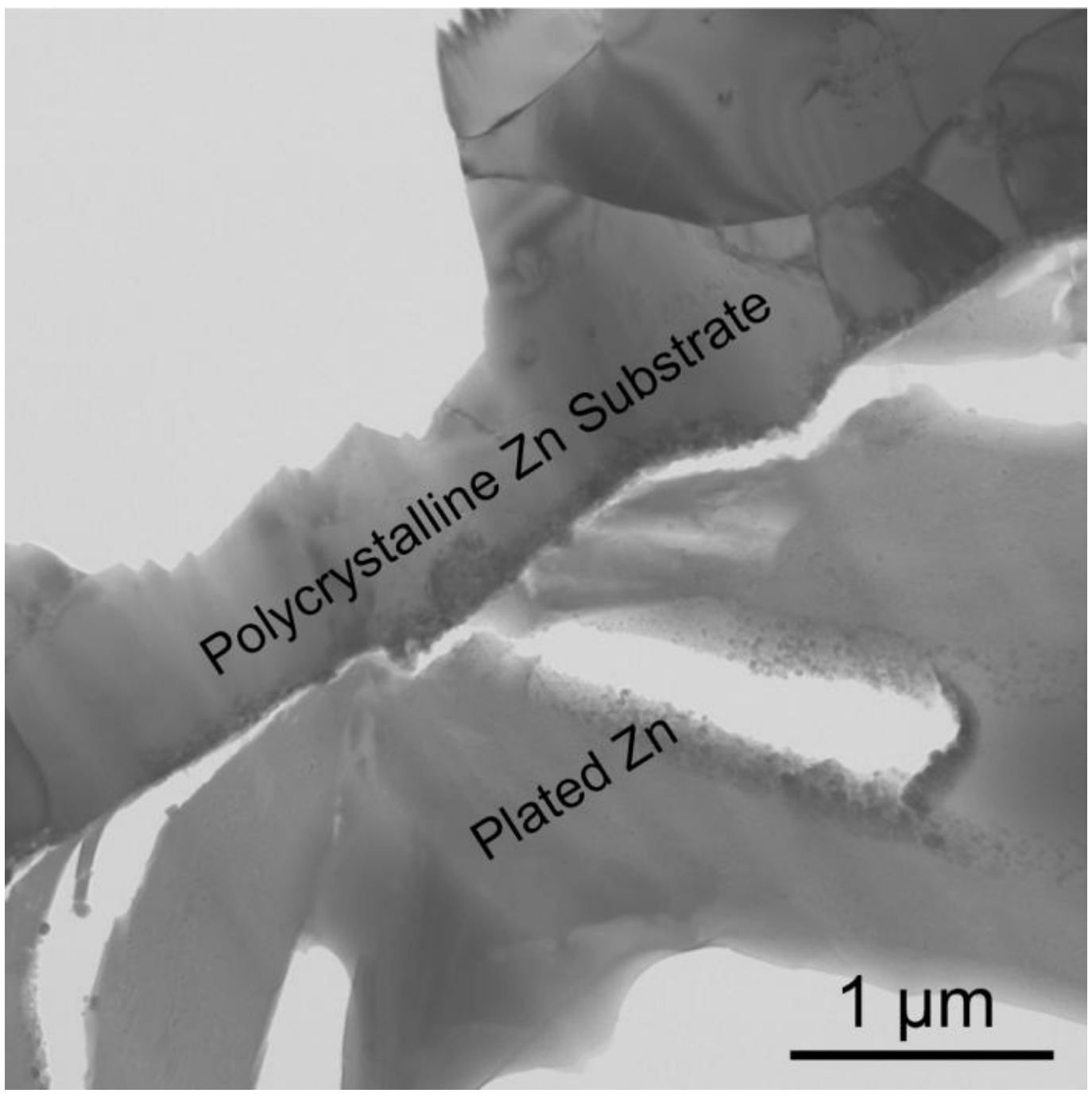

Figure S25. TEM image of plated Zn in ZS electrolyte.

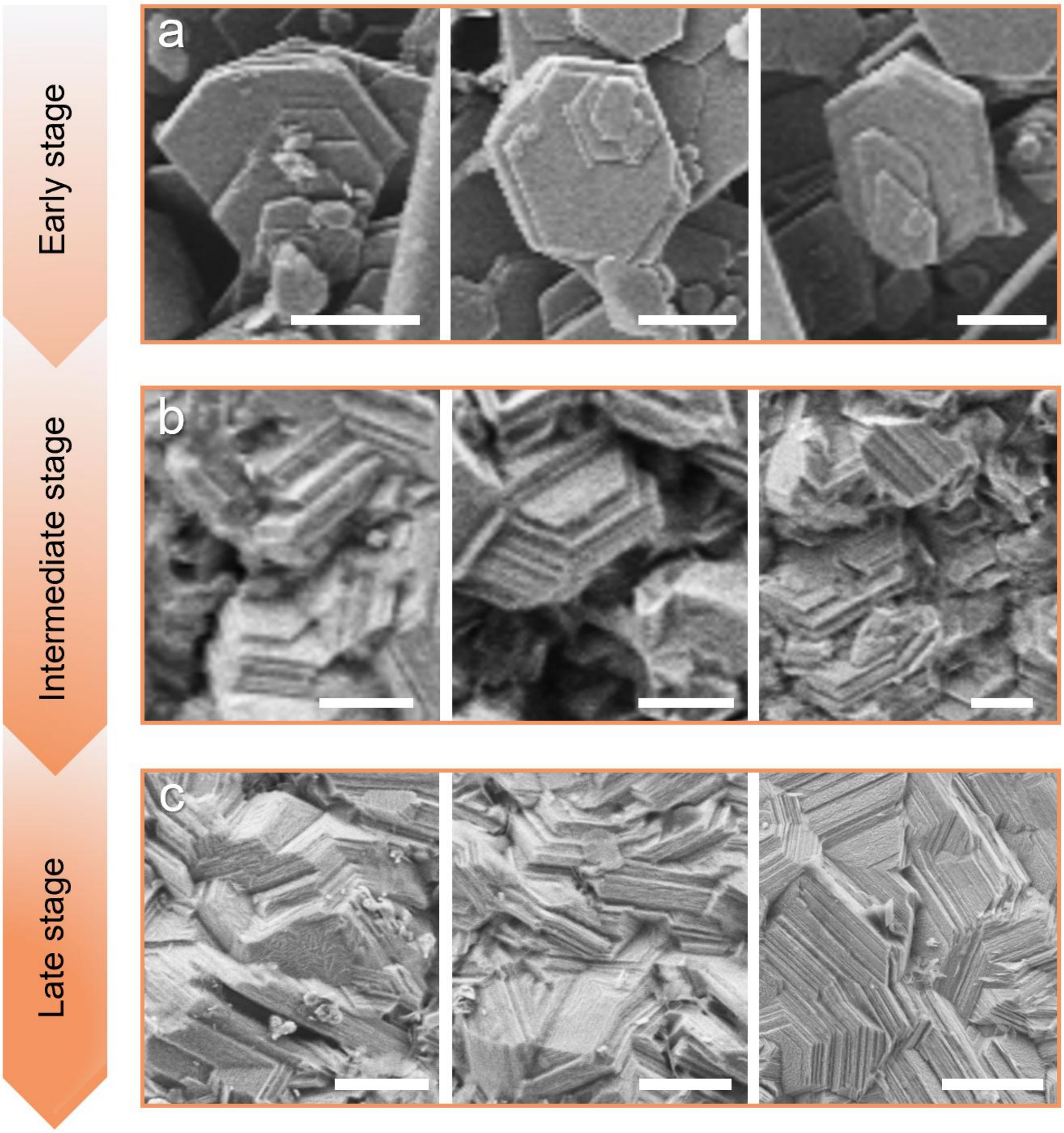

Figure S26. a-c) Screw-dislocation driven surface morphology evolution (early, intermediate, and late stages) of Zn plated in DZS electrolyte at 1 mA cm^{-2} with a capacity of 1 mAh cm^{-2} (scale bars: 5 μm).

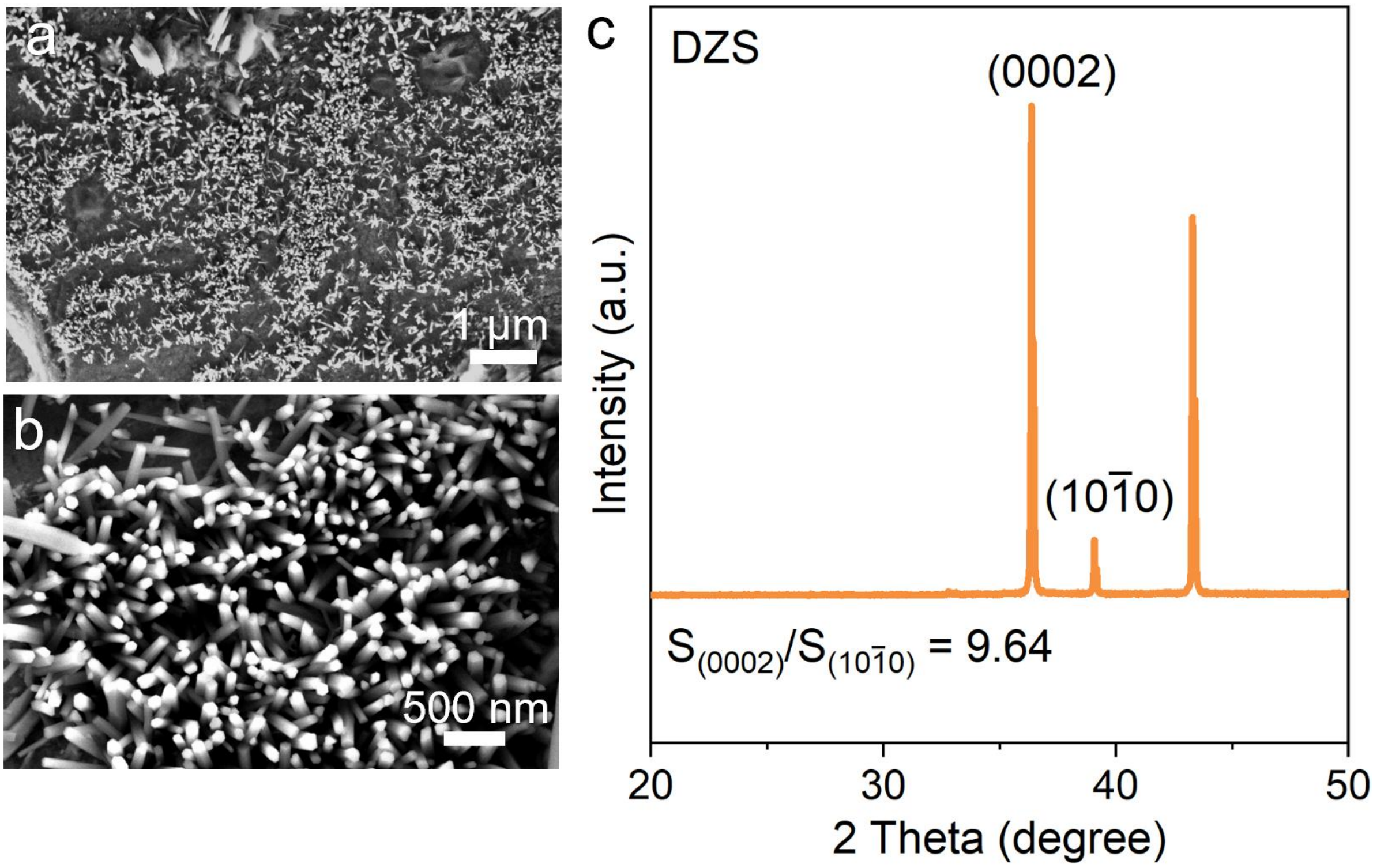

Figure S27. SEM images (a, b) and XRD pattern (c) of the plated Zn in electrolyte with 0.5 M $DyCl_3$ additive.

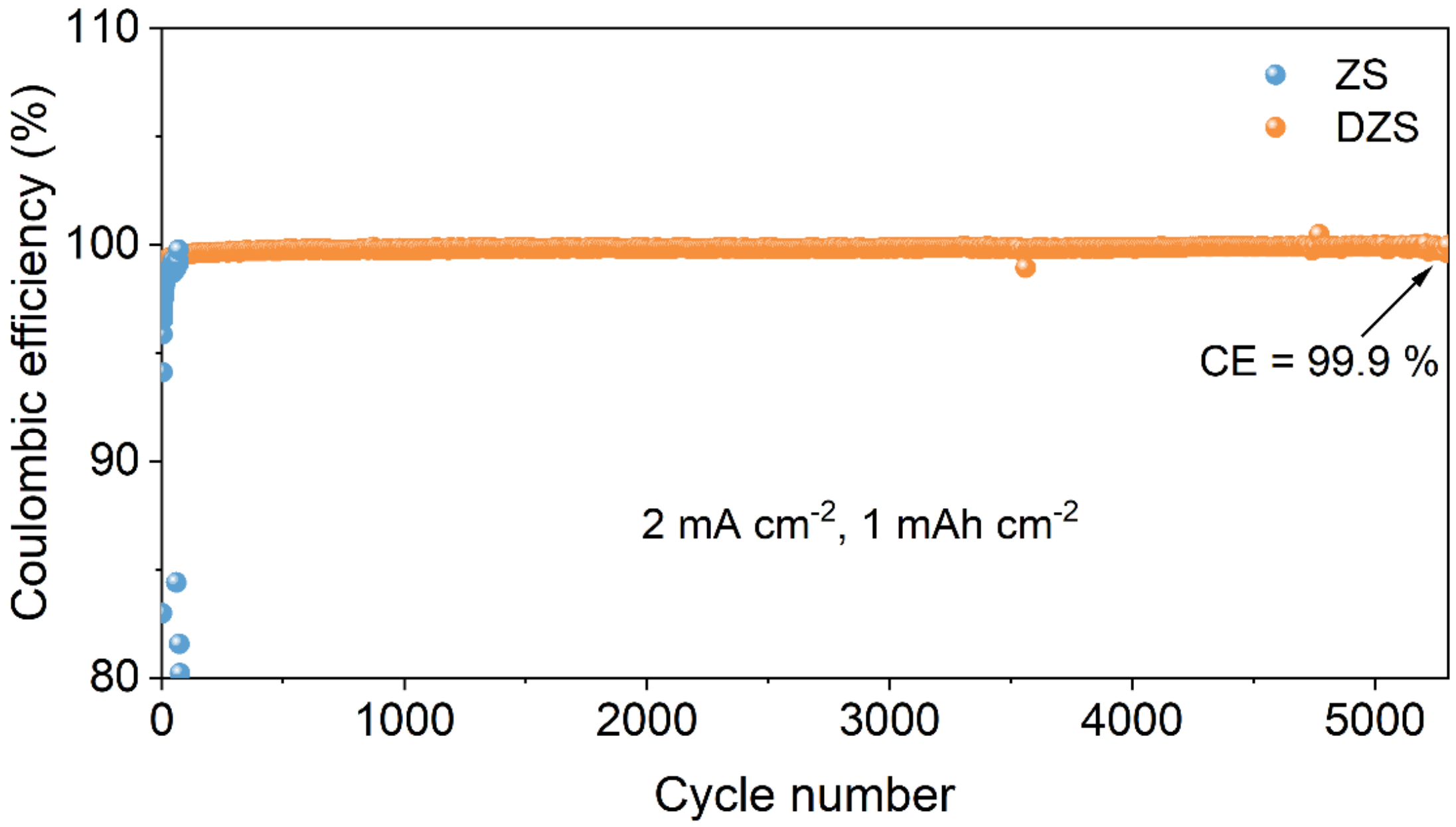

Figure S28. CE at 2 mA cm^{-2} with a capacity of 1 mAh cm^{-2} of Zn||Cu cells with ZS and DZS electrolytes, respectively.

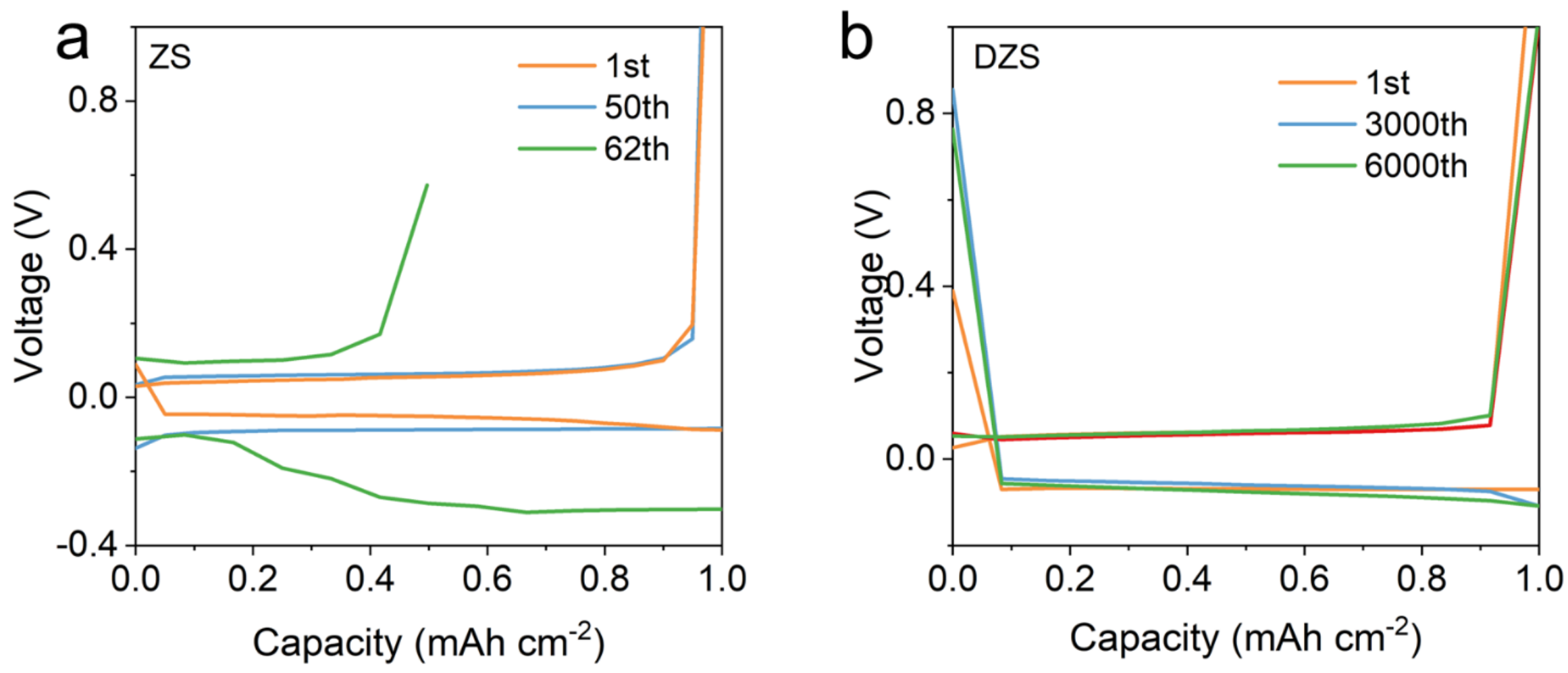

Figure S29. GCD curves of Zn||Cu cells at 10 mA cm^{-2} with a capacity of 1 mAh cm^{-2} in ZS (a) and DZS (b) electrolytes.

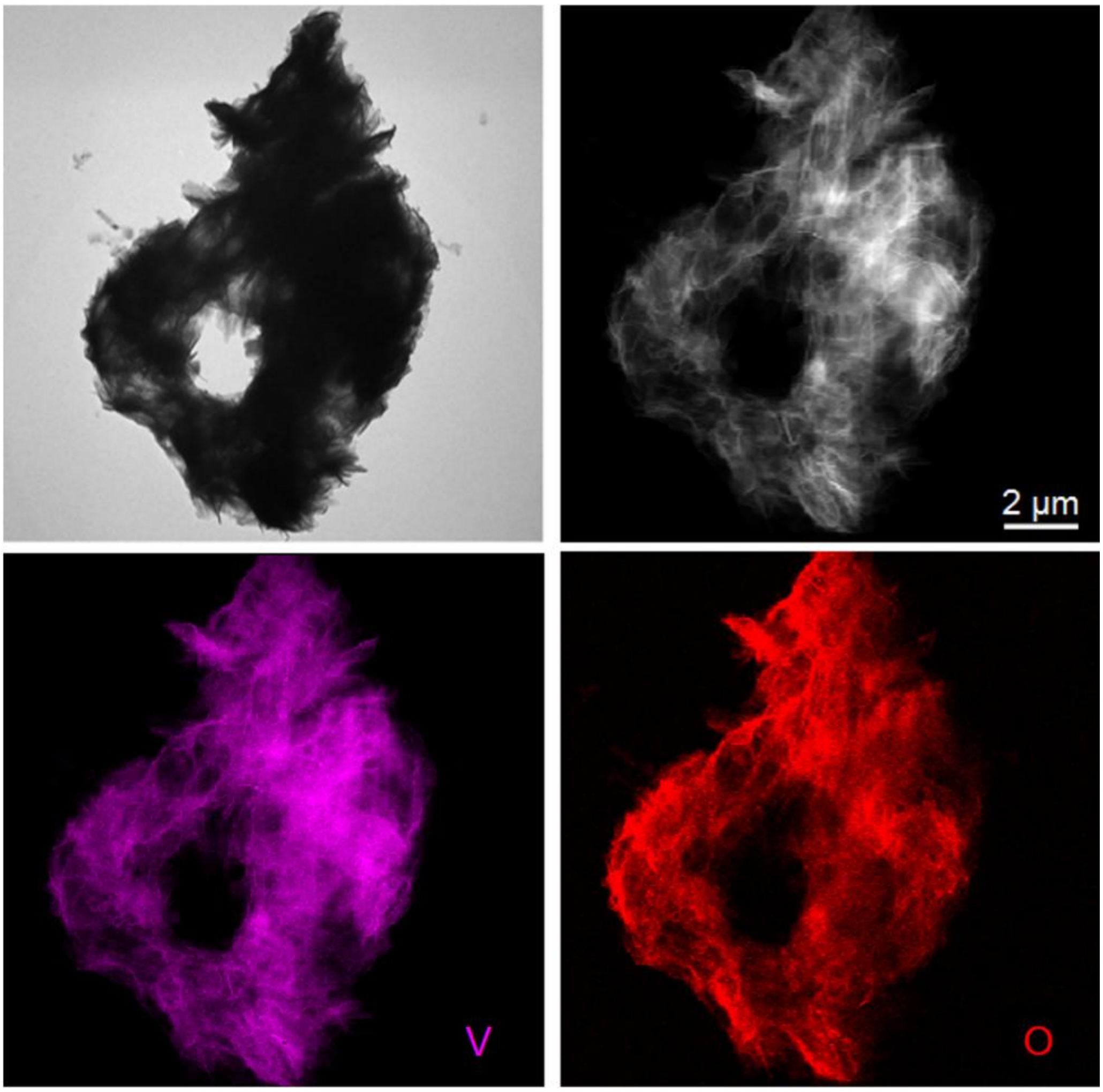

Figure S30. TEM image and EDS element mappings of V_2O_5.

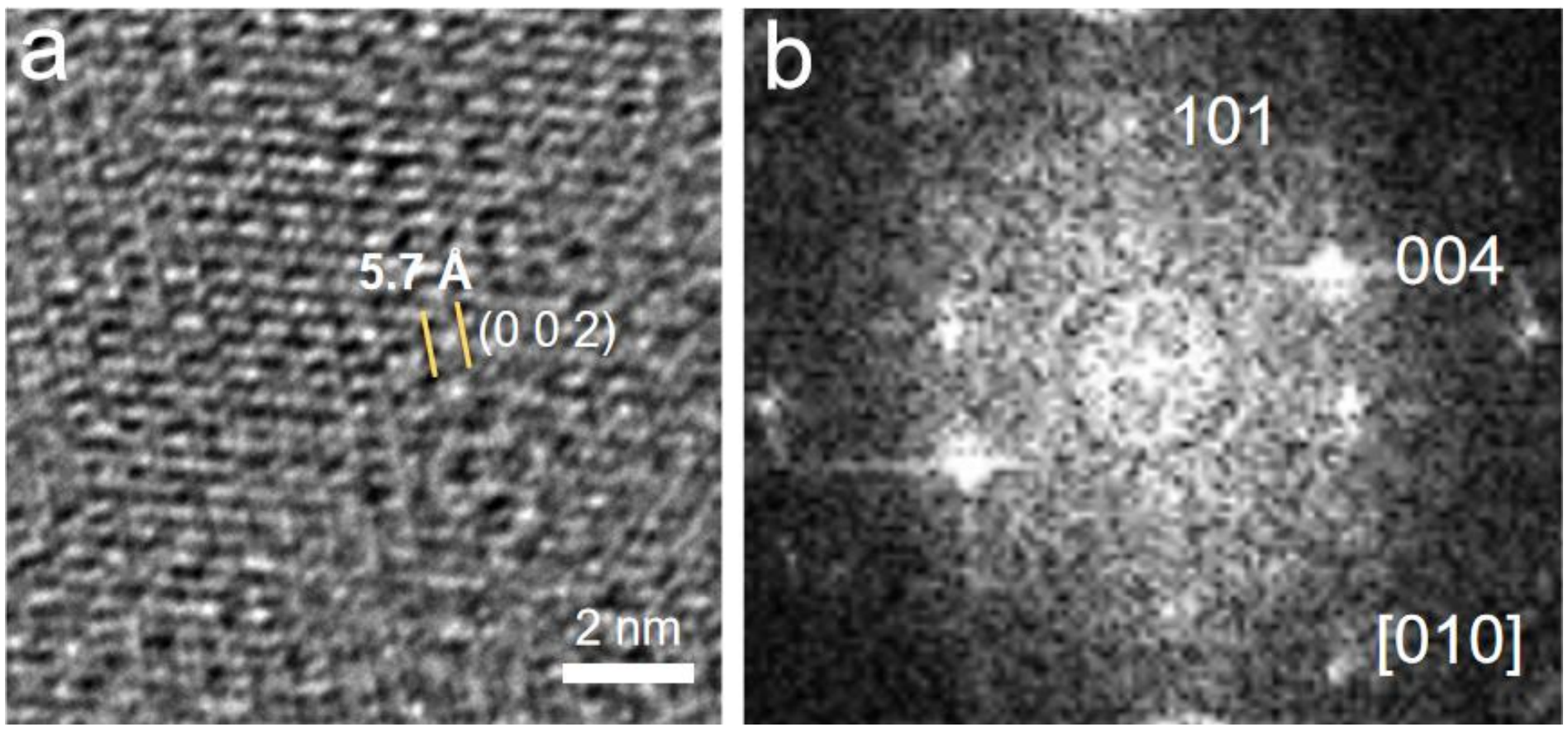

Figure S31. HRTEM image (a) and corresponding fast Fourier transform (FFT) diagram (b) of V_2O_5.

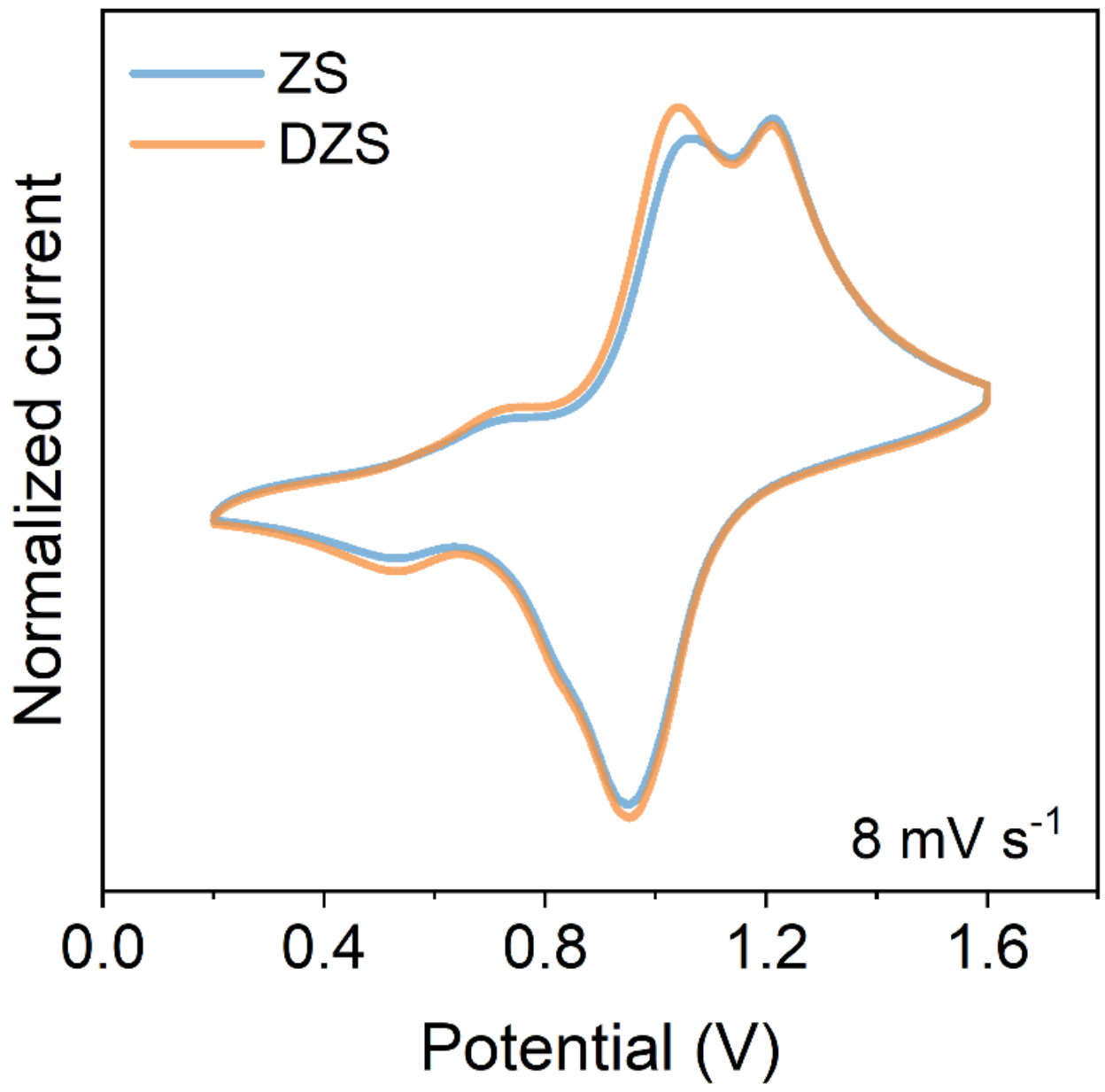

Figure S32. CV curves of the Zn||V_2O_5 cells in ZS and DZS electrolytes.

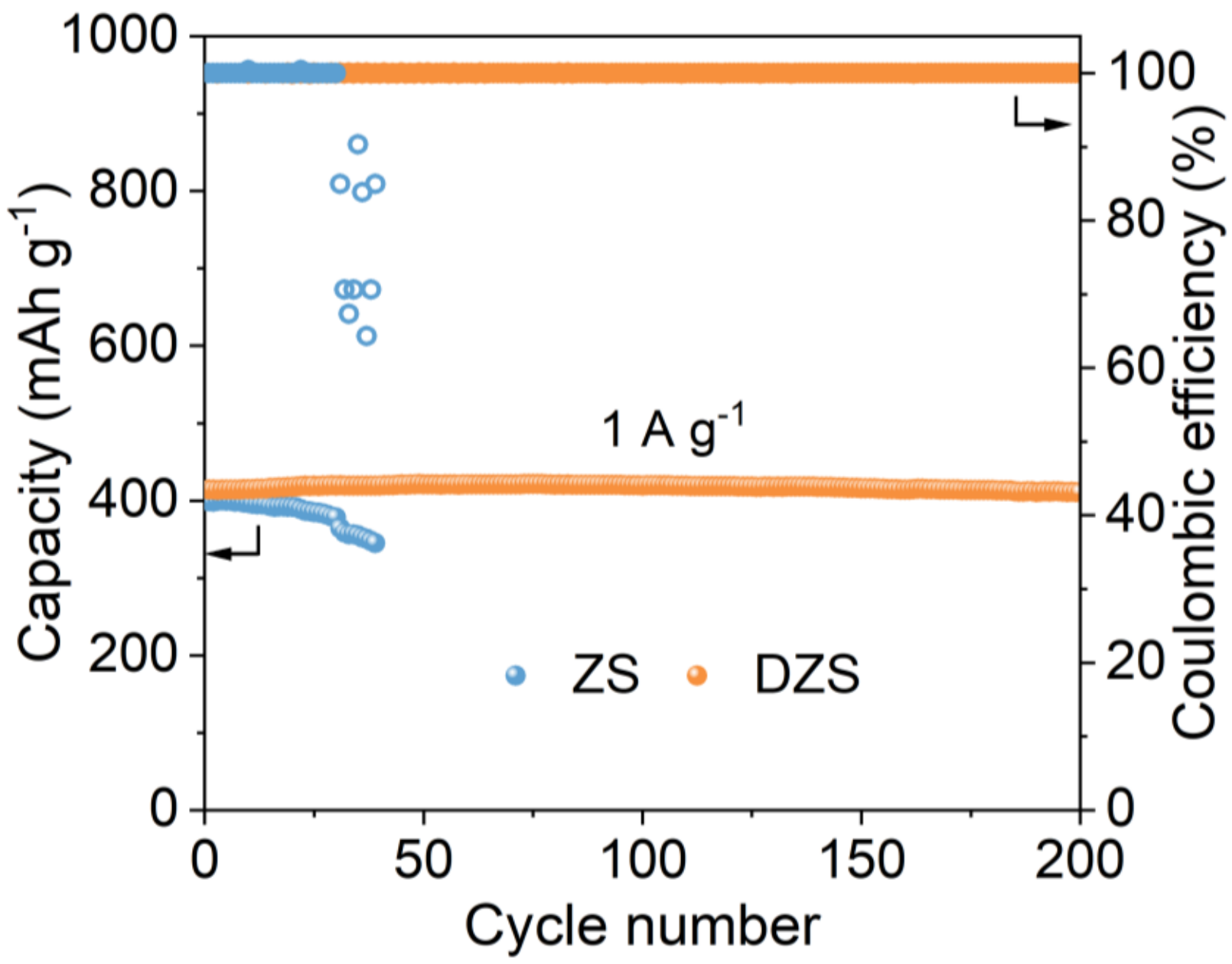

Figure S33. Cycling performance at 1 A g^{-1}.

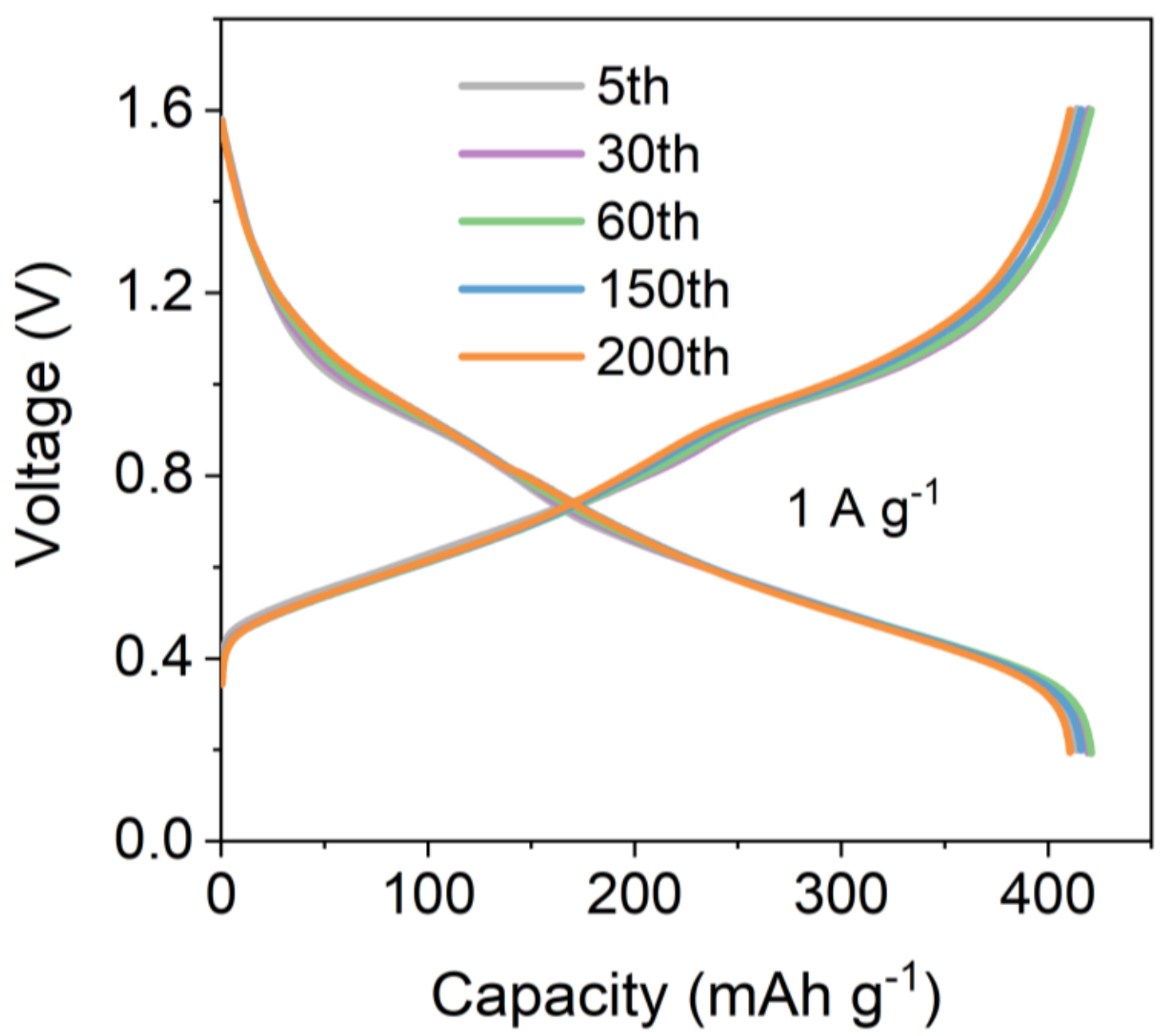

Figure S34. GCD curves of Zn||V_2O_5 cell in DZS electrolyte.

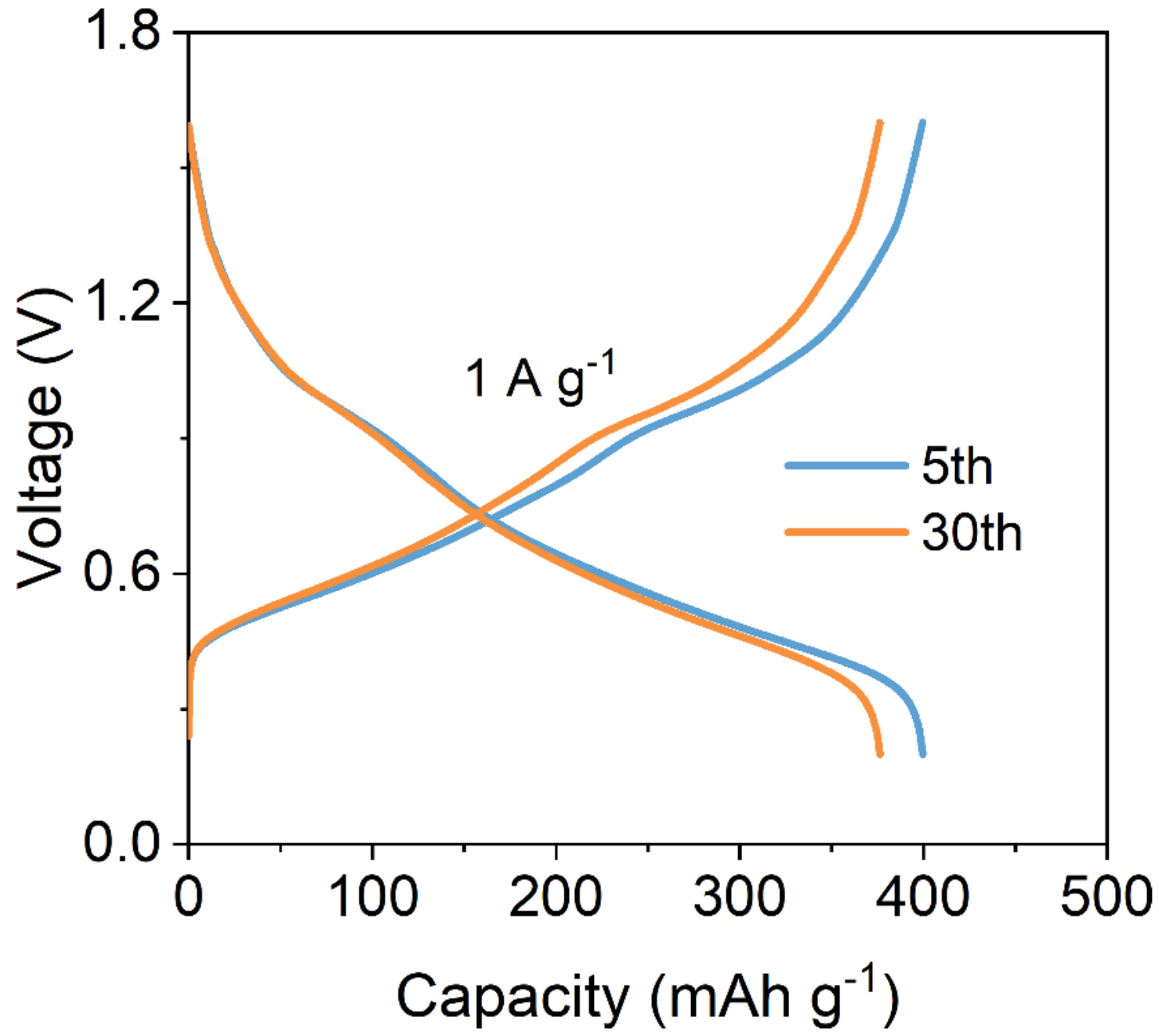

Figure S35. GCD curves of Zn||V_2O_5 cell in ZS electrolyte.

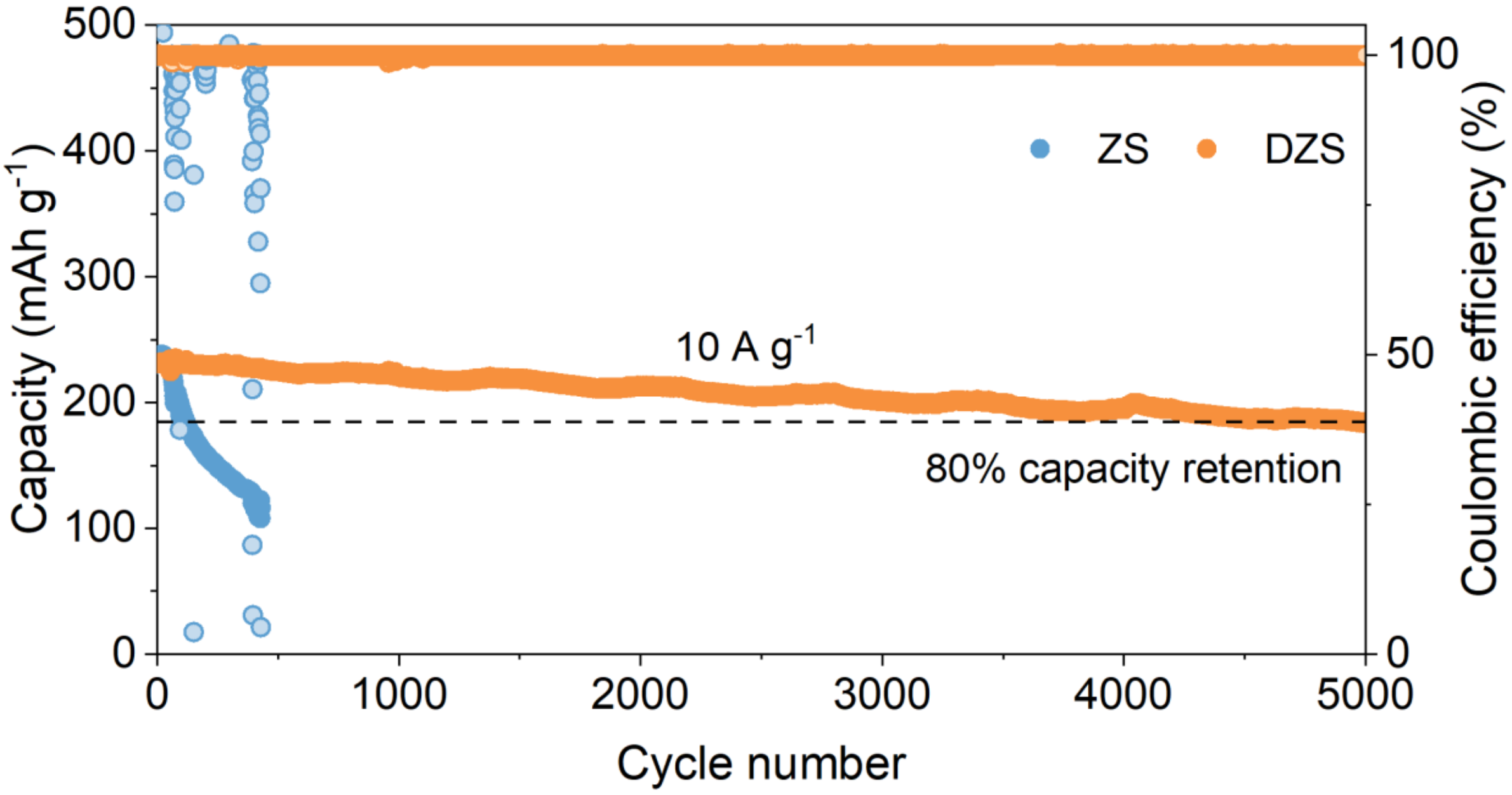

Figure S36. Long-term cycling performance at 10 A g^{-1}.

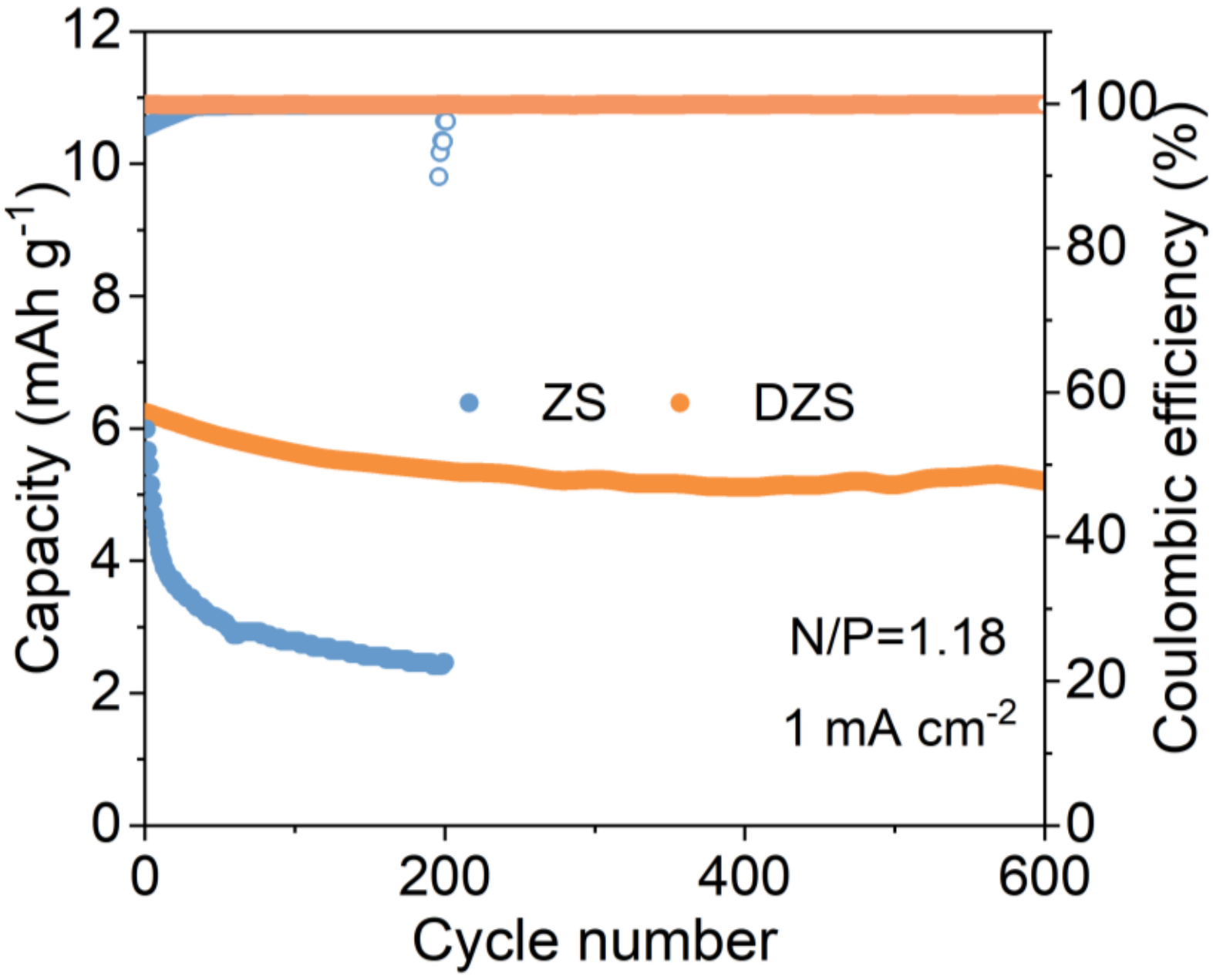

Figure S37. Cycling performance (1 mA cm^{-2}) of pouch-type full cells with a low N/P capacity ratio of 1.18.

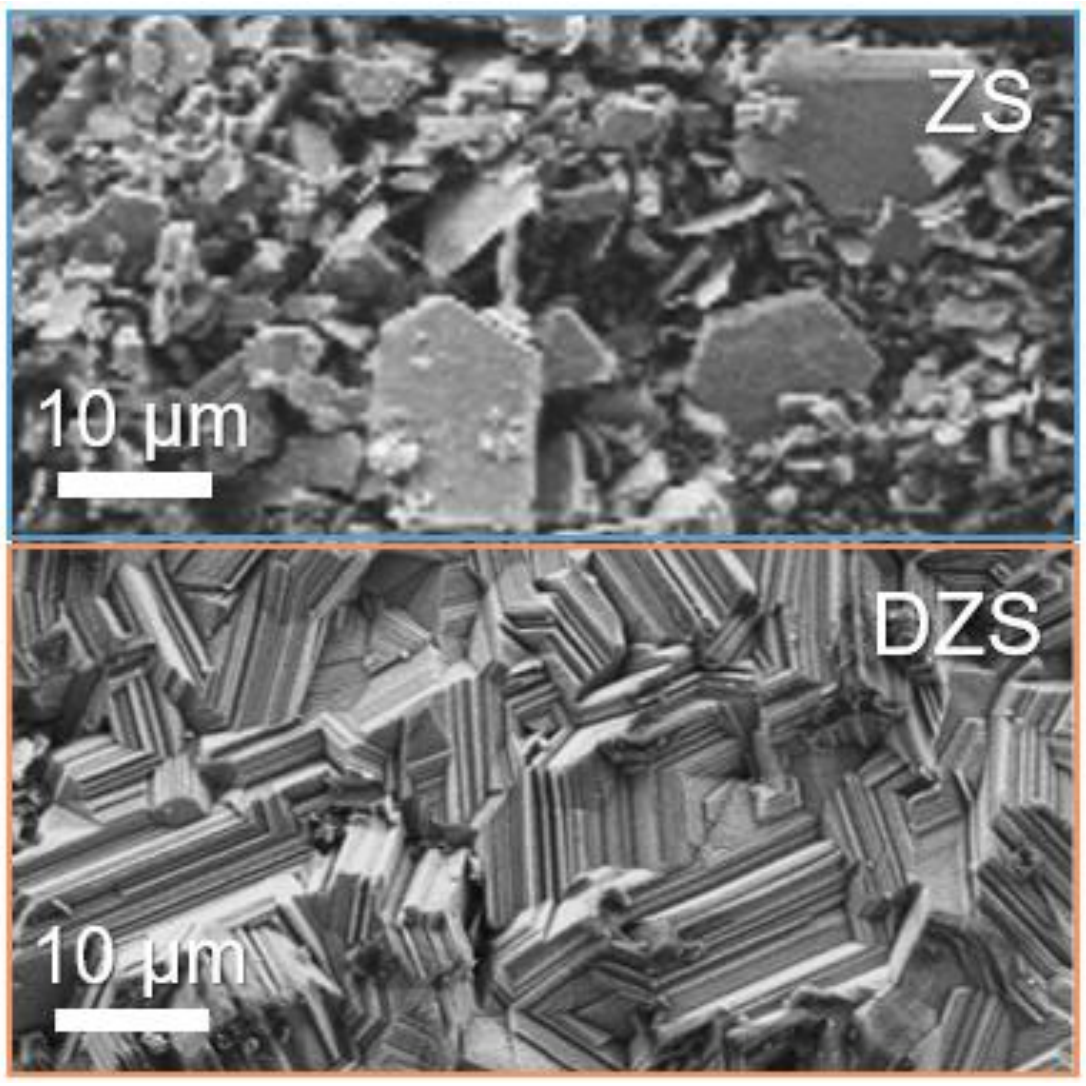

Figure S38. SEM images of cycled Zn anodes after long-term operation in ZS and DZS electrolytes, respectively.

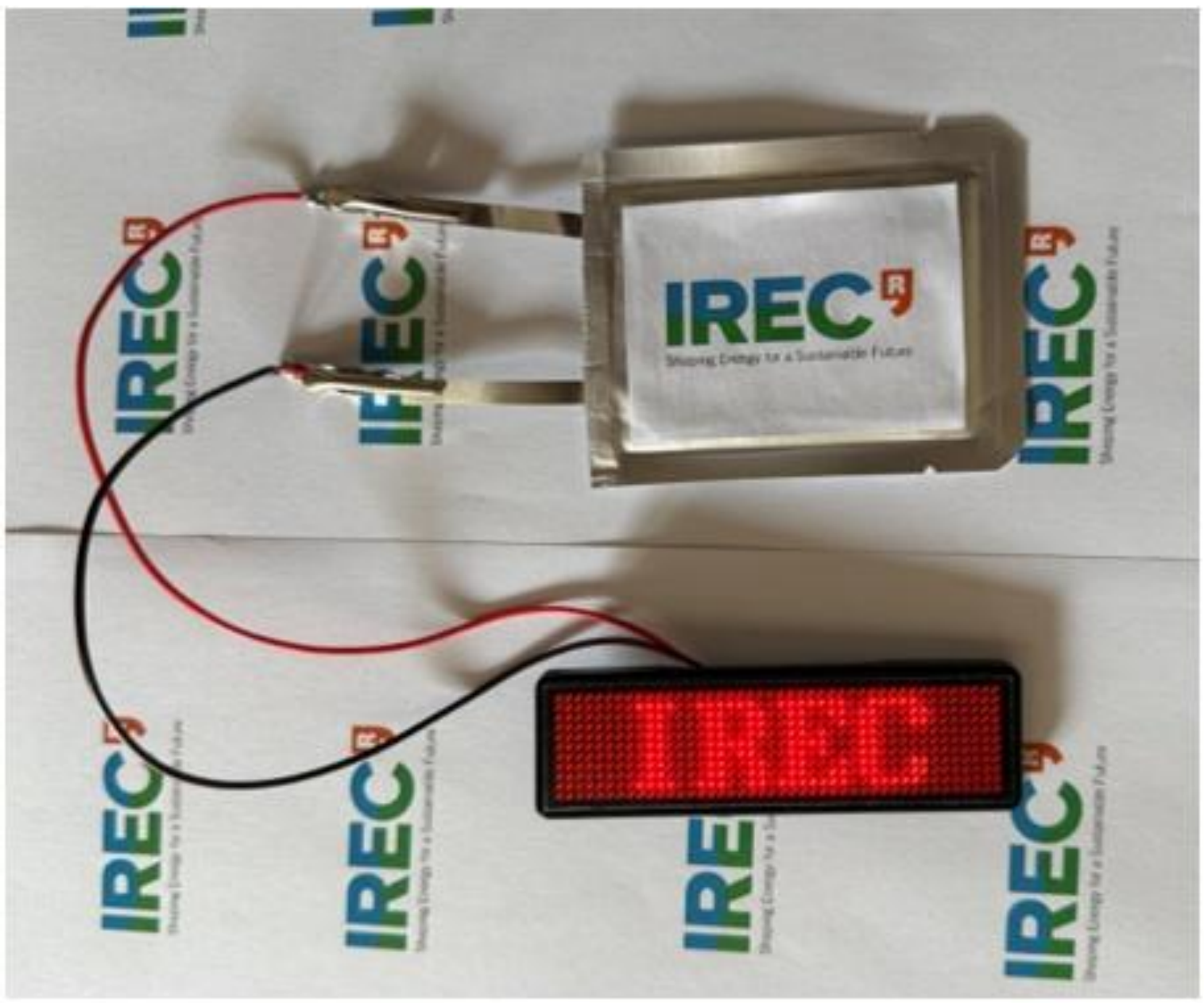

Figure S39. Photograph of a pouch-type cell powering an LED display.

Table S1. The E_{corr} and i_{corr} of the ZS and DZS electrolytes.

Sample	i_{corr} (mA cm^{-2})	Percentage decrease in i_{corr}	E_{corr} (V)
ZS	2.06	/	-0.972
DZS_1	1.73	16%	-0.958
DZS_2	1.75	15%	-0.960
DZS_3	1.71	17%	-0.958
DZS_4	1.73	16%	-0.957
DZS-0.1	0.42	80%	-0.936

Table S2. Fitting parameters of Stern–Geary cross check.

Cross-check of Tafel plots in Figure 1g					
Sample	R_p (V/mA cm^{-2})	β_a (V/dec)	β_c (V/dec)	i_{corr} (mA cm^{-2}) Stern-Geary method	i_{corr} (mA cm^{-2}) Extrapolation method
ZS	0.0185	0.183	0.170	2.06	2.06
DZS	0.0211	0.164	0.180	1.77	1.73
Percentage decrease in i_{corr}				14%	16%

Table S3. Parameter comparisons with reported Zn||Zn symmetric batteries.

Electrolyte	CPC (Ah cm^{-2})	Time (h)	Current density (mA cm^{-2})	Reference
DZS	15.00	3000	10	This work
TFA	5.50	1100	10	[2]
ZSF	4.60	4600	2	[9]
DZS	4.50	150	60	This work
DZS	4.25	8500	1	This work
Sr^{2+}+ZSO	3.50	3500	2	[8]
TFA	3.40	1360	5	[2]
ZSO/Ce	3.40	170	40	[6]
PS-2.2	3.00	2000	3	[4]
d-SEI	2.80	5600	1	[14]
ZSO + BED	2.63	525	10	[3]
Ga^{3+}+ZSO	2.40	4000	1.2	[7]
[EMIM]OTF/$ZnSO_4$	2.25	900	5	[5]
ZSO+CA+APM	2.25	4500	1	[13]
ZSO + BED	1.75	3150	1	[3]
ZSO-CoSA/C	1.00	2000	1	[10]
La^{3+}-ZS	0.81	160	10	[12]
1Z-5H	0.79	20	79	[11]
BPE	0.60	6000	0.2	[15]

References

[1] M. Stern, A. L. Geary, *J. Electrochem. Soc.* **1957**, 104, 56.

[2] M. Wu, X. Wang, F. Zhang, Q. Xiang, Y. Li, J. Guo, *Energy Environ. Sci.* **2024**, 17, 619-629

[3] X. Ren, G. Chen, P. Chang, S. Ju, Y. Wu, *Energy Environ. Sci.* **2025**, *18*, 1867-1878.

[4] H. Yang, L. Li, D. Chen, J. Wang, Y. Tan, Z. Jiang, Y. Zhang, C. Miao, W. Zhang, W. Han, G. He, *Angew. Chem. Int. Ed.* **2025**, *64*, e202419394.

[5] J. Weng, W. Zhu, K. Yu, J. Luo, M. Chen, L. Li, Y. Zhuang, K. Xia, Z. Lu, Y. Hu, C. Yang, M. Wu, Z. Zou, *Adv. Funct. Mater.* **2024**, *34*, 2314347.

[6] Z. Hu, F. Zhang, Y. Zhao, H. Wang, Y. Huang, F. Wu, R. Chen, L. Li, *Adv. Mater.* **2022**, *34*, e2203104.

[7] J. Cao, Y. Jin, H. Wu, Y. Yue, D. Zhang, D. Luo, L. Zhang, J. Qin, X. Yang, *Adv. Energy Mater.* **2025**, *15*, 2403175.

[8] J. Cao, J. Wu, H. Wu, Y. Jin, D. Luo, X. Yang, L. Zhang, D. Zhang, J. Qin, J. Lu, *Adv. Funct. Mater.* **2024**, *34*, 2401537.

[9] G. Zeng, Q. Sun, S. Horta, P. R. Martínez-Alanis, P. Wu, J. Li, S. Wang, M. Ibáñez, Y. Tian, L. Ci, A. Cabot, *Energy Environ. Sci.* **2025**, *18*, 1683-1695.

[10] W. Fan, P. Li, J. Shi, J. Chen, W. Tian, H. Wang, J. Wu, G. Yu, *Adv. Mater.* **2024**, *36*, 2307219.

[11] M. Kim, J. Lee, Y. Kim, Y. Park, H. Kim, J. W. Choi, *J. Am. Chem. Soc.* **2023**, *145*, 15776–15787.

[12] R. Zhao, H. Wang, H. Du, Y. Yang, Z. Gao, L. Qie, Y. Huang, *Nat. Commun.* **2022**, *13*, 3252.

[13] T. Xue, Y. Mu, Z. Zhang, J. Guan, J. Qiu, C. Yang, L. Zang, L. Zeng, *Adv. Energy Mater.* **2025**, 2500674.

[14] M. Zhao, Y. Lv, Y. Xu, H. Yang, Z. Bo, J. Lu, *Nat. Commun.* **2025**, *16*, 2843.

[15] J. Zhu, M. Yang, Y. Hu, M. Yao, J. Chen, Z. Niu, *Adv. Mater.* **2024**, *36*, 2304426.
